# Scanning superlens microscopy for non-invasive large field-of-view visible light nanoscale imaging

**DOI:** 10.1038/ncomms13748

**Published:** 2016-12-09

**Authors:** Feifei Wang, Lianqing Liu, Haibo Yu, Yangdong Wen, Peng Yu, Zhu Liu, Yuechao Wang, Wen Jung Li

**Affiliations:** 1 State Key Laboratory of Robotics, Shenyang Institute of Automation, Chinese Academy of Sciences, Shenyang 110016, China; 2 University of Chinese Academy of Sciences, Beijing 100049, China; 3 Department of Mechanical and Biomedical Engineering, City University of Hong Kong, Kowloon Tong 999077, Hong Kong

## Abstract

Nanoscale correlation of structural information acquisition with specific-molecule identification provides new insight for studying rare subcellular events. To achieve this correlation, scanning electron microscopy has been combined with super-resolution fluorescent microscopy, despite its destructivity when acquiring biological structure information. Here we propose time-efficient non-invasive microsphere-based scanning superlens microscopy that enables the large-area observation of live-cell morphology or sub-membrane structures with sub-diffraction-limited resolution and is demonstrated by observing biological and non-biological objects. This microscopy operates in both non-invasive and contact modes with ∼200 times the acquisition efficiency of atomic force microscopy, which is achieved by replacing the point of an atomic force microscope tip with an imaging area of microspheres and stitching the areas recorded during scanning, enabling sub-diffraction-limited resolution. Our method marks a possible path to non-invasive cell imaging and simultaneous tracking of specific molecules with nanoscale resolution, facilitating the study of subcellular events over a total cell period.

## Abstract (web summary)

Rare subcellular events can be tracked by correlating structural-information gathered by imaging with specific-molecule fluorescent identification. Here, the authors achieve this correlation in a quick and non-invasive way using microsphere-based scanning superlens microscopy.

To fully explore the basic mechanisms in life and material sciences, and in other areas, research has gradually moved into the nanoscale using various novel microscopy technologies. Traditional electron microscopy, which achieves a molecular- and atomic-level resolution, has played an irreplaceable role in this transition; however, the radiation dose that the specimens are exposed to by the imaging system’s energetic electron beam is comparable to the irradiation from the explosion of a 10-megaton hydrogen bomb ∼30 m away, which confines the application of electron microscopy to non-destructive cell observation^1^,^2^. Scanning probe microscopies provide another convenient approach to obtain sub-nanometre resolution in both air and liquid, but their invasiveness may induce unrecoverable structural damage or morphology deformation in sensitive specimens. Specifically, the interaction forces they produce may drive adherent cells to detach from a dish surface during scanning with atomic force microscopy (AFM). The adhesion between cells and tips causes cell damage and limits the reusability of the probe and potentially contributes to cross-contamination. Recently, advancements in life studies have accelerated markedly due to the development of super-resolution fluorescence microscopes that circumvent the optical diffraction limit induced by the loss of evanescent waves in far-field imaging, producing nanoscale resolutions^3^,^4^,^5^. Fluorescent labelling could allow the identification of specific molecules and to explore the functions of these molecules in life^4^,^6^; however, this labelling also induces a loss of structural information because a majority of molecules are unlabeled^7^. The implementation of a large fraction of these imaging methods depends on a point-based raster scanning technology, such as the commonly used scanning electron microscopy (SEM)^8^, scanning probe microscopy (SPM)^9^,^10^ and stimulated emission depletion (STED)^11^ microscopy. The acquisition time increases significantly as the imaging area (for example, >50 × 50 μm^2^) is enlarged at a high resolution (for example, <100 nm). In addition, SPM and SEM typically use a slow scanning speed and increase the number of scan lines used to improve imaging quality^12^, which further leads to time inefficiencies. To increase the imaging speed, parallelized scanning systems have been designed for different types of microscopes (for example, SEM^13^, SPM^14^,^15^ and STED^16^,^17^), making the systems more complex and expensive, and requiring sophisticated algorithms to achieve system control and image processing^18^. Only few of these systems are commercially available currently. Compared with these microscopes, traditional optical microscopes have certain unique advantages, such as the ability for non-invasive, real-time, large-area and fluorescent or white-light imaging, but their resolution is limited by the diffraction limit. Recently, the diffraction barrier has been surpassed by simply introducing dielectrics with a micro-scale spherical configuration when using conventional optical microscopes by transforming evanescent waves into propagating waves^18^,^19^,^20^,^21^,^22^,^23^,^24^,^25^,^26^,^27^,^28^,^29^,^30^. The resolution of this superlens-based microscopy has been decreased to ∼50 nm (ref. 26) from an initial resolution of ∼200 nm (ref. 21). This method can be further enhanced to ∼25 nm when coupled with a scanning laser confocal microscope^31^. It has achieved fast development in biological applications, as the sub-diffraction-limited resolution of high-index liquid-immersed microspheres has now been demonstrated^23^,^32^, enabling its application in the aqueous environment required to maintain biological activity. Experimental results have also shown potential applications in biological observations in both fluorescent^29^,^30^ and white-light^25^ modes. This method inherits the majority of the advantages of traditional optical microscopes; however, the imaging field of view (FOV) is confined by the microsphere superlens’ size and by the aberrations in the boundary area of the FOV. Although the FOV increases linearly with the diameter of the superlens, the resolving ability deteriorates as the diameter increases^23^. This limited FOV reduces the range of practical applications; therefore, methods, such as probe-based manipulations^18^,^21^ and immersing microspheres into elastomers^19^,^20^,^28^, have been reported to locate microsphere superlenses for observations from a specific position. Unfortunately, when using these methods, it is difficult to achieve large-area imaging and to locate specific sub-diffraction-limited targets in a large area. In particular, these targets appear in different areas, such as the distribution of lysosomes in cells. In addition to these methods, microspheres or introduced elastomers still make contact with the samples, and the distance or interaction force between the microspheres and samples cannot be precisely controlled due to the lack of effective feedback mechanisms, which further adds to the invasiveness of this microsphere-based super-resolution imaging method.

In the following, we introduce a non-invasive, environmentally compatible and high-throughput optical microscopy technique called scanning superlens microscopy (SSUM) for large-area, super-resolution imaging and structural information acquisition. The distance or interaction forces between the microsphere superlenses and the specimens are adjusted using AFM principles, allowing the scanning and imaging of large areas. On the basis of the microsphere superlens imaging properties, two different AFM scanning modes that are compatible with microsphere superlens imaging have been demonstrated to achieve microsphere scanning of a large area: the contact mode enables imaging of stiff samples, and the constant-height mode facilitates non-invasive imaging of sensitive specimens. Compared with point-based raster scanning microscopes, this new technique replaces the ‘point’ with an ‘area’ (that is, the middle area of the FOV of the microsphere superlens has no aberrations); therefore, there is a significant improvement in terms of the time efficiency for large-area imaging.

## Results

### Description of the microsphere-based SSUM

**Figure 1:**
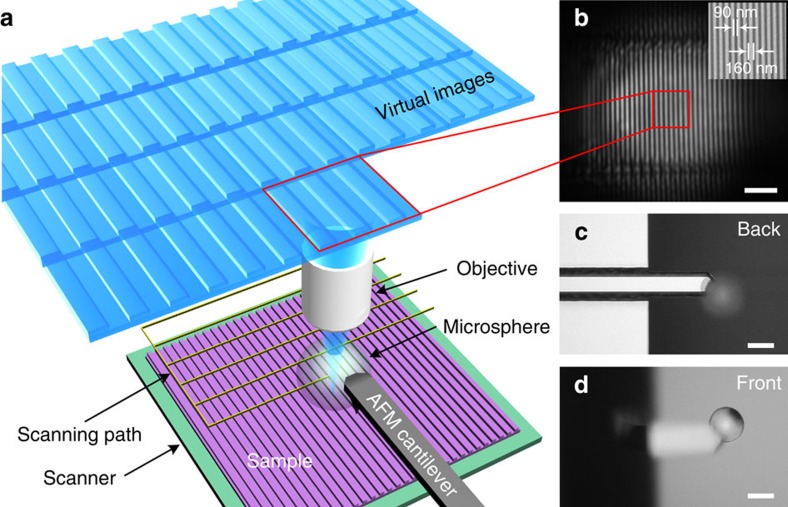
Microsphere-based SSUM. (**a**) Schematic of the construction of a microsphere-based SSUM that integrates a microsphere superlens into an AFM scanning system by attaching the microsphere to an AFM cantilever. The objective picks up the virtual images containing sub-diffraction-limited object information and simultaneously focuses and collects the laser beam used in the cantilever deflection detection system. (**b**) An original virtual image observed using the microsphere superlens. The inset shows an SEM image. (**c**,**d**) Backside and frontside images, respectively, of the AFM cantilever with an attached microsphere superlens. Scale bars, 2 μm (**b**); 50 μm (**c**,**d**).

Figure 1a shows the basic concept of a microsphere-based SSUM. An AFM distance or interaction force control mechanism is introduced to adjust the position of the microsphere above the sample. The magnification of the microscope objectives used in the microsphere superlens-based nanoscale imaging is typically > × 50 with a limited working distance^18^,^22^,^23^,^24^,^25^,^26^. To be compatible with these objectives, a custom AFM system (Supplementary Fig. 1) illuminates and collects the laser beam of the optical lever using the same objective^33^,^34^. The microsphere superlens is attached to an AFM cantilever (Fig. 1c,d) that is fixed during scanning to prevent the distance between microsphere and the objective from changing or to maintain the imaging conditions as much as possible. Raster scanning in the lateral directions and feedback regulation in the vertical direction are achieved using a three-dimensional piezoelectric ceramic (PZT) scanner on which the specimens are placed. The spacing of the raster scanning and the interval of the signal used to trigger camera image recording during lateral scanning are adjusted based on the area of the FOV of microsphere superlenses without apparent aberration (Fig. 1b and Supplementary Fig. 2). Large-area, super-resolution imaging can be achieved by stitching together the recorded images. Here ‘super-resolution’ is defined as resolutions higher than *λ*/3.8 by considering the solid-immersion effect^35^,^36^, and the resolution of SSUM mounted with a 57 μm-diameter BaTiO_3_ microsphere is estimated to be *λ*/6.3 under partial and inclined illumination, where *λ* is the peak illumination wavelength (Supplementary Note 1 and Supplementary Figs 3 and 4). The resolution could be further improved by the application of smaller microspheres^23^.

### Contact scanning mode

**Figure 2:**
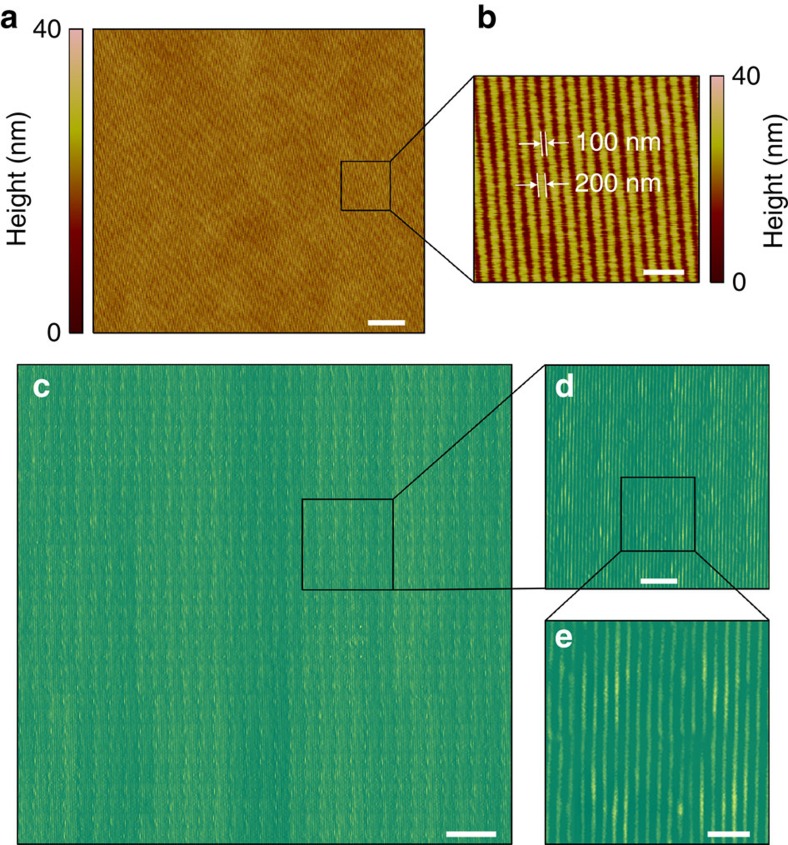
Large-area fast imaging in contact scanning mode. (**a**) AFM scanning image of a Blu-ray disk surface. (**b**) Zoom-in of **a**. (**c**) Large-area imaging using the SSUM in contact scanning mode. (**d**) Zoom-in of **c**. (**e**) Zoom-in of **d**. In this study, a × 50 (numerical aperture=0.6) objective was used. Scale bars, 10 μm (**a**,**c**); 1 μm (**b**,**e**); 3 μm (**d**).

Considering the near-field imaging characteristic of the microsphere superlens, we first demonstrate the capability of large-area imaging by the scanning microsphere using the contact mode. In this study, contact mode indicates that the microsphere instead of the AFM probe tip is in contact with the sample during scanning. To achieve this and to sufficiently scan the image (1) the microsphere diameter should be greater than the tip height if there is a tip on the AFM probe, or an AFM probe without tip should be selected particularly when the microsphere diameter is small. If the microsphere is smaller than the tip (that is, the tip is in contact with the sample and the microsphere is suspended over the sample), it is also possible to achieve super-resolution imaging, but the position of microspheres on the cantilever must be precisely controlled. (2) The microsphere diameter and the cantilever spring constant should be properly selected to prevent vibrations during scanning. For a commercially available cantilever (for example, the TESP probe from Bruker), the microsphere diameter should be <80 μm, which is a practical conclusion. (3) The selection of the microsphere size should balance the FOV and the resolving ability^23^. (4) The cantilever does not shield the light path for the microsphere imaging (Fig. 1c,d). Figure 2c–e shows the large-area imaging of a Blu-ray disc surface, which contains 200 nm wide stripes spaced by 100 nm groves, that was achieved by scanning with a barium titanate (BaTiO_3_) microsphere with a diameter of 59 μm mounted on a TESP probe cantilever in water. The minimum feature size of the Blu-ray disc is ∼100 nm, which cannot be directly observed using an optical microscope. We defined three sample points in 100 nm to satisfy Nyquist criterion and resolve the minimum structure using a commercial AFM (Dimension Icon, Bruker). The total scanning area was ∼96 × 96 μm^2^; therefore, 2,880 scan lines were required for AFM scanning, and the real acquisition time was ∼4 h at a scan rate of 0.2 Hz (or 38.4 μm s^−1^) to guarantee imaging quality (Fig. 2a,b). However, only 32 lines of scanning were required when using the microsphere-based SSUM (that is, the imaging efficiency was increased × 90 compared with the traditional AFM method), and the acquisition time was ∼3 min. The pixel size for the image obtained by the SSUM was ∼14 nm. The AFM method required 6,857 scan lines to acquire the same image, which corresponds to ∼9.5 h; therefore, the SSUM is ∼ × 214 more efficient in terms of imaging.

### Constant-height scanning mode

**Figure 3:**
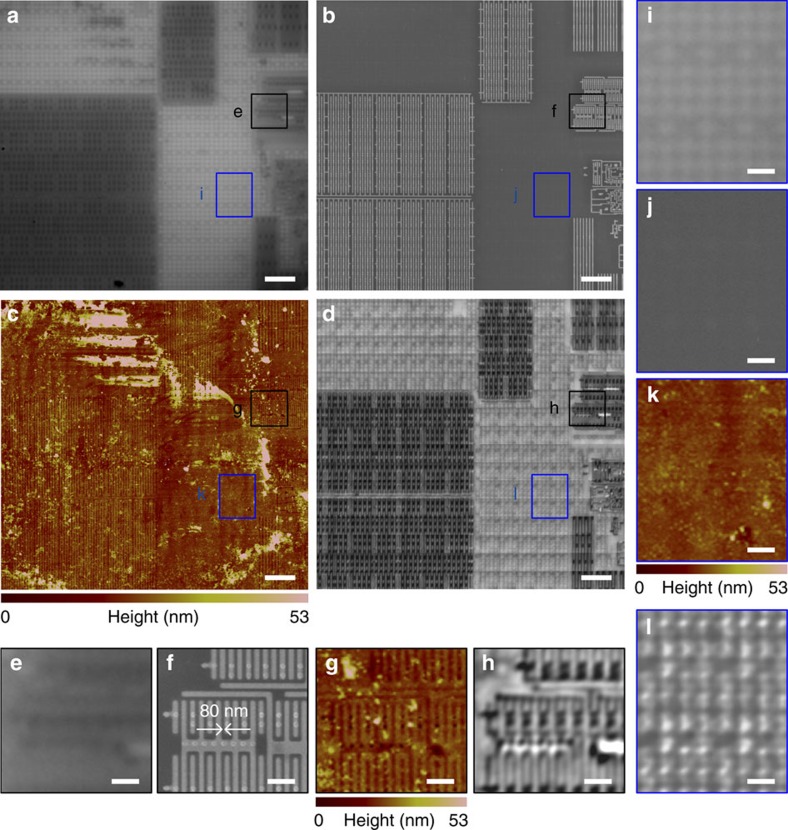
CPU sub-surface structure imaging in constant-height scanning mode. Comparison of (**a**) a conventional microscope mounted with a × 100 (numerical aperture=0.8) objective, (**b**) SEM, (**c**) AFM and (**d**) the SSUM for the observation of CPU sub-surface structures beneath a 10±2 nm (measured by AFM)-thick optically transparent film preventing AFM and SEM detection, which are denoted by **i**–**l**. (**e**–**l**) Local zoomed areas that correspond to the marked areas in **a**–**d**. Scale bars, 5 μm (**a**–**d**); 1 μm (**e**–**l**).

For sensitive specimens, such as cells, the interaction forces in contact mode will induce structural deformation or damage; therefore, a non-invasive method is essential. This non-invasive imaging technique can be achieved using the SSUM within conjunction with a fundamental AFM working principle: the constant-height mode that is commonly used to achieve atomic-resolution images^37^,^38^,^39^. In this mode, the microsphere is suspended over the samples, and the distance between the microsphere apex and the surface of the PZT scanner is held constant. We first examine the constant-height scanning mode by resolving structures in a central processing unit (CPU) that has specific structures of ∼80 nm over a large area by scanning with a 57 μm diameter BaTiO_3_ microsphere in water at a scan rate of 0.1 Hz. The scan rate is the same in the following experiments unless noted otherwise. A video recording of the scanning process is provided as Supplementary Movie 1, corresponding to the results shown in Supplementary Fig. 5d. There are certain structures or defects generated in the CPU sample preparation process that influence AFM imaging (Fig. 3c and Supplementary Fig. 5c), but are not apparently observed in the SEM images (Fig. 3b and Supplementary Fig. 5b). Some of these defects are also shown in the optical images (Fig. 3a and Supplementary Fig. 5a); however, they do not prevent the observation of structures in these areas. The influence of these defects on imaging is further weakened, and sub-diffraction-limited structures are resolved when imaged using the microsphere superlenses (Fig. 3d and Supplementary Fig. 5d) compared with the images obtained when they are directly observed using an optical microscope (Fig. 3a and Supplementary Fig. 5a). The virtual image magnification factors of the microsphere superlens in Fig. 3 and Supplementary Fig. 5 are both × 3.4. Compared with the features imaged in Supplementary Fig. 5, several structures are located in Fig. 3i–l that can be clearly observed only in the microsphere-enhanced optical images (Fig. 3l). One possible reason for these results is that there is an optically transparent film preventing the energetic electron beam and AFM probes from penetrating and properly imaging the sample^40^, but the film has a negligible influence on visible light transmission or microsphere superlens-based imaging, that is, the microsphere superlens can image sub-diffraction-limited structures below optically transparent films or realize sub-surface imaging.

**Figure 4:**
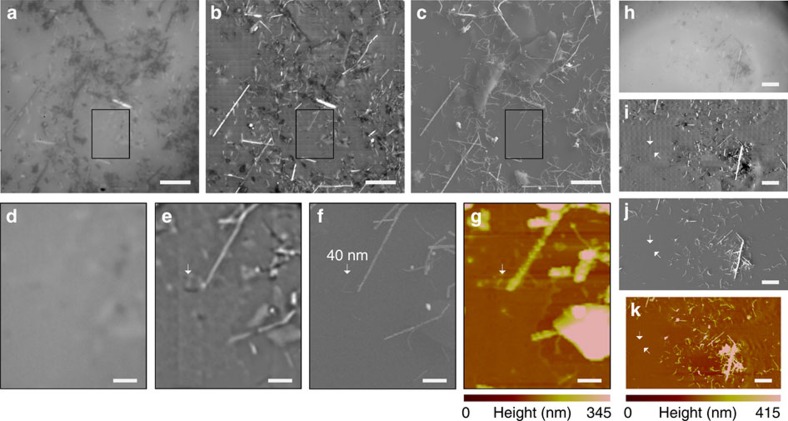
AgNW imaging in constant-height scanning mode. (**a**,**h**) AgNWs are directly observed using an optical microscope. (**b**,**i**) AgNWs imaged using a scanning microsphere superlens. A × 100 (numerical aperture=0.8) objective was used in these experiments. (**c**,**j**) SEM images. The arrow in **f** points to a AgNW with a diameter of 40 nm. (**g**,**k**) AFM scanning images. (**d**–**f**) Local zoomed areas that correspond to the marked areas shown in **a**–**c**, respectively. The corresponding AFM scanning image is shown in **g**. Scale bars, 10 μm (**a**–**c**,**h**–**k**); 2 μm (**d**–**g**).

**Figure 5:**
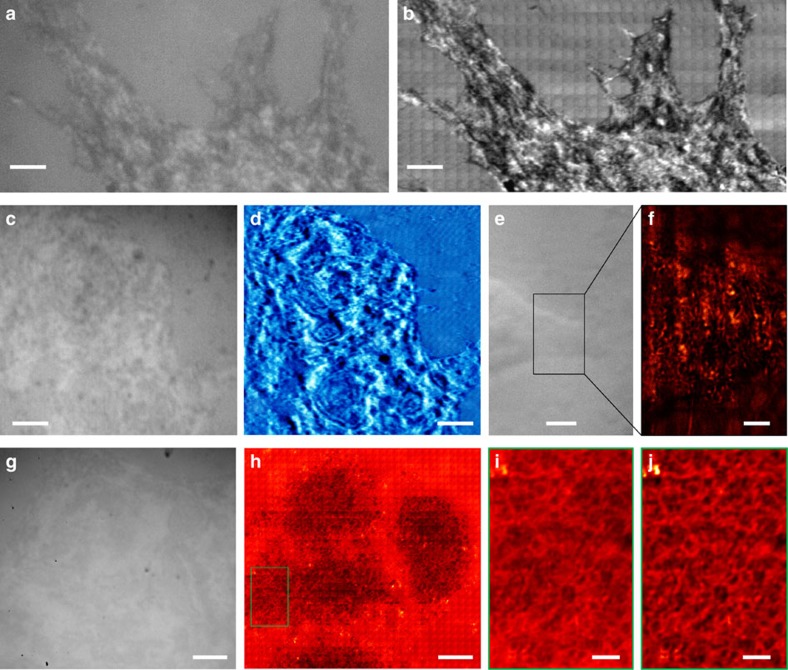
Non-invasive observation of cells in white-light mode. A C2C12 cell was imaged using (**a**) a traditional optical microscope or (**b**) SSUM. A video recorded while scanning a C2C12 cell is provided as Supplementary Movie 2. MCF-7 cells were observed (**c**,**e**,**g**) without and (**d**,**f**,**h**) with the aid of the microsphere superlens. A × 100 (numerical aperture (NA)=0.8) objective was used in **a**,**b**,**g** and **h**, and a × 50 (NA=0.6) objective was used in **c**–**f**. (**i**) Local zoomed area of the marked area shown in **h**. (**j**) After using a band-pass filter algorithm of **i**. Scale bars, 6 μm (**a**,**b**); 10 μm (**c**–**e**,**g**,**h**); 3 μm (**f**); 2 μm (**i**,**j**).

**Figure 6:**
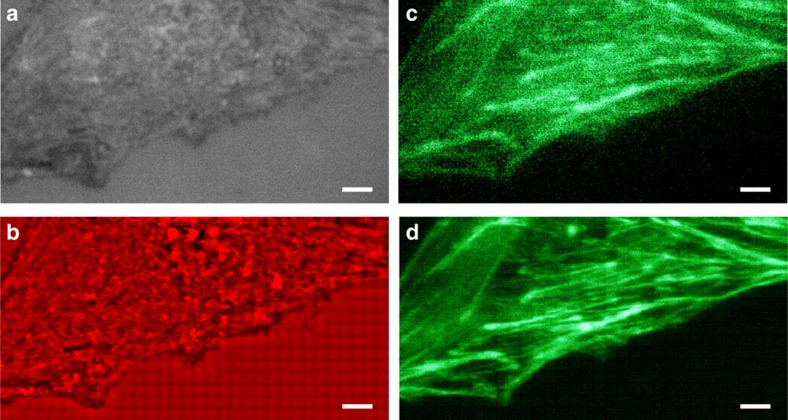
Non-invasive white-light and fluorescence microscopy of a C2C12 cell. (**a**,**b**) White-light and (**c**,**d**) fluorescent imaging of a C2C12 cell (**a**,**c**) without and (**b**,**d**) with the enhancement of a 56 μm-diameter microsphere superlens. A × 100 (numerical aperture=0.8) objective was used in these experiments. For fluorescent imaging, the sample was labelled by Alexa Fluor 488-phalloidin to observe actin filaments. Scale bars, 5 μm.

To further demonstrate the penetration ability of the microsphere superlens, we observe randomly distributed silver nanowires (AgNWs) covered by an ∼11 nm-thick, optically transparent polystyrene film by scanning with a 57 μm diameter BaTiO_3_ microsphere in water, as shown in Fig. 4. This scan was conducted using the constant-height scanning mode. With the assistance of the microsphere superlens, sub-diffraction-limited AgNWs covered by the transparent film were clearly resolved (Fig. 4b,i), and a 40 nm-diameter AgNW was located by SSUM in a large area (Fig. 4e). Figure 5 shows the non-invasive scan imaging of a mouse myoblast cell (C2C12) and human breast cancer cells (MCF-7) using constant-height mode. These scans were performed using a BaTiO_3_ microsphere superlens with a diameter of 59 or 63 μm in water. As shown in Fig. 5b,d, an improved surface morphology of cells could be captured. More detailed surface structural information can be resolved compared with the result directly observed using an optical microscope (Fig. 5a,c). In other experiments, we also discerned certain filaments using 55 or 50 μm-diameter BaTiO_3_ microspheres, as shown in Fig. 5f,h,i. The distribution of these structures does not correspond to the cell surface morphology, comparing the results shown in Fig. 5g,h, which differs from the cases shown in Fig. 5a–d. Because these filaments are not distributed across the cell membrane, they should exist inside the cell, which is similar to the results shown in Figs 3l and 4b,i; thus, sub-membrane nanostructures that reside inside live cells (Fig. 5f) or fixed cells (Fig. 5h) beyond the diffraction barrier can be non-invasively explored over a large area under white-light illumination. The sub-membrane nanostructures can also be resolved via SEM in hydrated samples assisted by graphene, but the cell must be fixed, membrane-extracted and stained, which causes cell death^41^. The image quality can be further improved using a band-pass filter algorithm (Fig. 5j). Because there are more than 10,000 types of proteins in one cell^25^, which form various functional units, it is difficult to distinguish objects with similar structures through only label-free optical observation, for example, the sub-diffraction-limited filaments observed in Figs 5f,h and 6b and in Supplementary Figs 6b,d and 7b. In contrast, fluorescent labelling could target specific molecules and provide a method to identify or locate specific structures. Here we identified actin filaments from cell structural information by comparing white-light and fluorescent images observed via SSUM (Fig. 6 and Supplementary Figs 7 and 8). These correlated analyses based on the white-light and fluorescent imaging provide a method of studying the detailed protein composition of filaments, as shown in Fig. 5f,h and in Supplementary Figs 6 and 7.

### Imaging characteristics of the microsphere superlens

**Figure 7:**
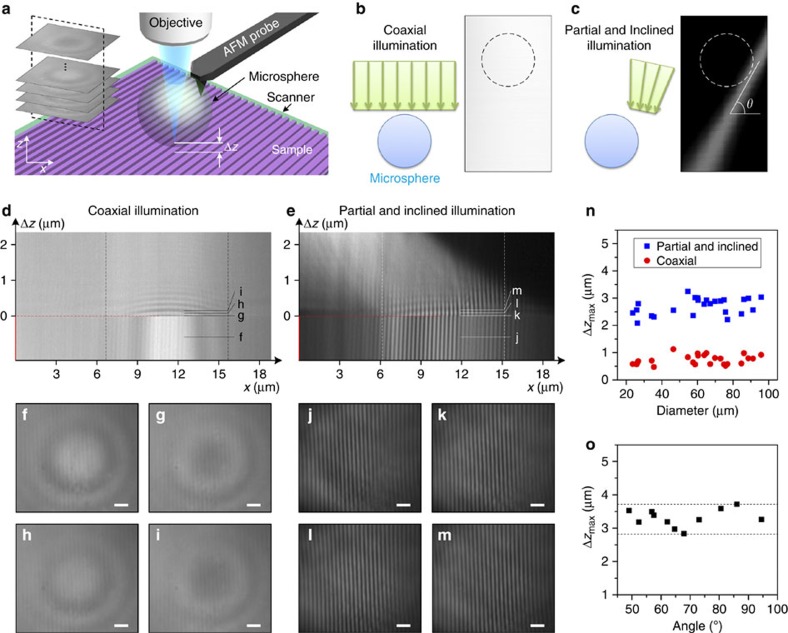
Microsphere imaging properties analyses. (**a**) Schematic showing the experimental set-up used to study the distance (Δ*z*) between the microsphere apex and the samples (Blu-ray disc) on microsphere imaging properties. The inset shows the image stack recorded during scanning. The left schematics in **b** and **c** show two different illumination conditions: coaxial illumination; and partial and inclined illumination. The right images in **b** and **c** show the illumination conditions detected during the vertical scanning of an optical beam profiler around the objective focus, where *θ* is the incline angle of the incident light focused using a × 50 (numerical aperture=0.6) objective. (**d**,**e**) Cross-sections of the images recorded during PZT scanning, as shown in the inset of **a**, under different illumination conditions. (**f**–**i**,**j**–**m**) Super-resolution images corresponding to the specific positions marked in **d** and **e**, respectively. (**n**) Relationship between Δ*z*_max_ and the microsphere diameter, where Δ*z*_max_ is the maximum distance that allows for imaging, that is, the structure on sample cannot be observed through the microsphere superlenses as Δ*z* exceeds Δ*z*_max_. The incline angle is set to ∼64° for partial and inclined illumination. (**o**) The influence of the inclined angle (*θ*) on Δ*z*_max_. The microsphere diameter is ∼60 μm in these measurements. Scale bars, 1 μm (**f**–**m**).

For traditional AFM constant-height imaging, the tip-sample distance is typically set to be sub-nanometre, and feedback is switched off. Therefore, scanning is confined to a limited area, which is typically measured in nanometres^37^,^38^,^39^. To elaborate why the microsphere superlenses can achieve a large scanning area in constant-height mode without feedback, we explored the influence of the separation distance (Δ*z*) between the microsphere and samples on microsphere super-resolution imaging (see Methods). In this study, the Blu-ray disc was held by the PZT scanner, vertically scanning away from the microsphere at different illumination conditions (Fig. 7b,c), which were recorded by a camera (inset of Fig. 7a). Figure 7d,e show the cross-sections of the recorded images; a Δ*z* value of zero indicates that the sample surface is in contact with the microsphere, and a Δ*z* value <0 indicates deformation towards the objective at the end of the cantilever. These results demonstrate that (1) limited marginal deformation of the cantilever towards the microscope objective (that is, Δ*z*<0 μm) does not induce apparent changes in imaging when the microsphere superlens is in contact with the sample; and (2) microsphere superlenses retain their imaging ability even when the microsphere is separated from the samples with sub-diffraction-limited structures (that is, Δ*z*>0 μm) as long as there is a peak wavelength of incident illumination (∼550 nm) for coaxial illumination and five wavelengths for partial and inclined illumination (Fig. 7n) that can be adjusted by the incline angle (Fig. 7o).

**Figure 8:**
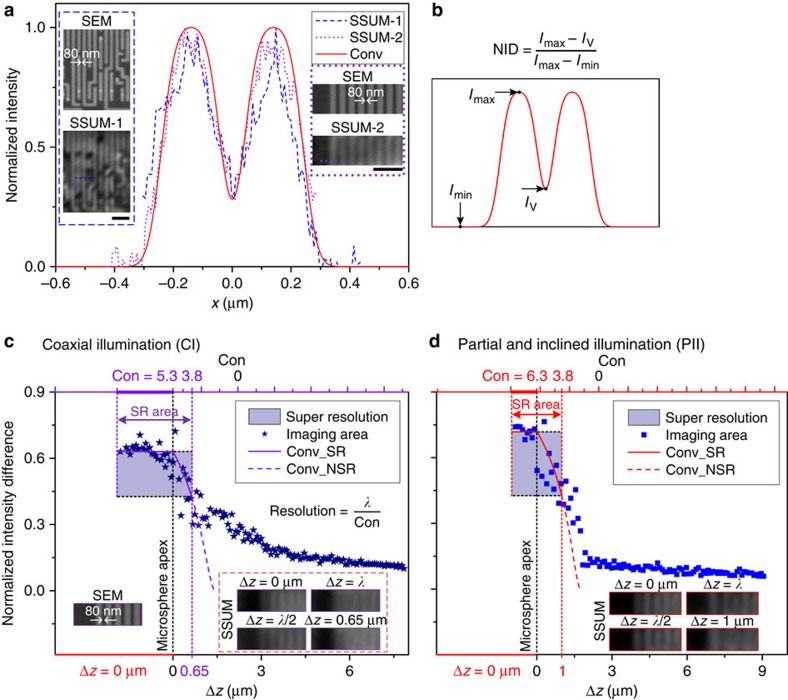
Resolution calibration of SSUM. (**a**) Insets are two sets of SEM images and SSUM results of the structures in the CPU, in which the dashed line (SSUM-1) and dotted line (SSUM-2) are used to estimate the resolution. The solid curve represents the calibrated convolution result. (**b**) Definition of NID. (**c**,**d**) The influence of Δ*z* on the resolution under different illumination conditions, that is, (**c**) coaxial illumination and (**d**) partial and inclined illumination. Δ*z* is defined in Fig. 7a, and the distance between microsphere and microscope objective does not change. The constants (Con) are the values applied to calculate the resolution (*λ*/Con). The data used to calculate NID (scatter data) are extracted from the experimentally measured cross-sections of the structures on the CPU chip (see the SEM image in the inset of **c**) using a 60 μm-diameter microsphere similar to that in Fig. 7d,e. In **c** and **d**, the solid curves, representing super-resolution, and dashed curves, representing the diffraction confinement, are achieved from the calibrated convolution results, for example, the solid curve in **a**. The incline angle, as defined in Fig. 7c, was set to ∼70° for the experiments under partial and inclined illumination. Scale bars, 1 μm (**a**).

Because the image generated by a microscope is the convolution of a point-spread function (PSF) and the object intensity distribution function, the resolution of SSUM can be estimated by a process of convolution with the PSF^22^,^35^,^36^. Using one-dimensional rectangular functions corresponding to two experimental results with minimum feature structures of 80 nm (insets of Fig. 8a), the resolution of SSUM mounted with a 57 μm microsphere is calibrated as the full-width at half-maximum, *λ*/6.3, of the Gaussian PSF according to Houston’s criterion^22^ (Fig. 8a, Supplementary Fig. 3 and Supplementary Note 1). This process is achieved by matching the calculated convolution results to the experimental profiles. A dimensionless parameter, normalized intensity difference (NID=(*I*_max_−*I*_V_)/(*I*_max_−*I*_min_); Fig. 8b), is defined to study the influence of Δ*z* on the resolution of microsphere superlenses under different illumination conditions. The NID calculated by experimental data (scatter data) and by the matched convolution results (solid or dashed curves) with different constants (Con) that are used in resolution estimation (resolution=*λ*/Con) are shown in Fig. 8c,d. The resolving capability of microspheres improves as Con increases. The resolution is attenuated (or the Con is decreased) with Δ*z* (Fig. 8c,d, Supplementary Fig. 4a,b and Supplementary Note 1), and the imaging quality decreases markedly as Δ*z* exceeds half of the wavelength for coaxial illumination and one wavelength for partial and inclined illumination, as shown in Fig. 7d,e. Therefore, Δ*z* should be controlled to be <Δ*z*_max_/2 to achieve high-quality imaging. This arrangement was used in the experiments performed in this study and facilitated the constant-height scanning mode of the microsphere superlens over a large area. The maximum separation between the microsphere and sample that allows for the super-resolution capability of a 60 μm microsphere is ∼0.65–0.8 μm for coaxial illumination and 1–1.7 μm for partial and inclined illumination, with degrading resolutions of *λ*/5.3 and *λ*/6.3 to *λ*/3.8, respectively (Supplementary Note 1). However, the imaging quality is deteriorated significantly at these separations since the evanescent waves with higher spatial frequencies will be quickly attenuated below the background noise level within one wavelength. That is, only the evanescent waves with lower spatial frequencies can transmit several wavelengths^42^. The distances between the microsphere apex and samples are ∼330 nm for the cases shown in Fig. 5f,h and ∼0.6 μm for the case shown in Fig. 5b,d. This difference is the primary reason why the surface morphology and sub-membranes of cells can be observed (Fig. 5).

As the spherical lens shrinks to the micro-scale, the relationship between the focal length and diameter/Δ*z* (Supplementary Figs 9 and 10) and the dependence of the virtual image magnification on Δ*z* (Supplementary Fig. 11) deviate from the approximation of geometrical optics. To describe the relationship between the near-field magnification and Δ*z*, we present a fitting formula based on the magnification factor calculated by the finite-difference time-domain (FDTD) method simulated focal lengths (*f*_FDTD_), *M*≈*k* × *f*_FDTD_/(*f*_FDTD_−(*D*/2+Δ*z*)), which yields a good match when *k*≈1.2 (Supplementary Fig. 11 and Supplementary Note 2). After demonstrating that the sample reflectivity has a negligible effect on the analyses (Supplementary Fig. 12 and Supplementary Note 3), we determined that the noise signal plays a significant role in the sub-diffraction-limited imaging but has a negligible influence on the imaging of structures larger than the diffraction limit (Supplementary Fig. 13). For microsphere-based super-resolution imaging, the converted propagation signal carrying sub-diffraction-limited structural information decays exponentially with Δ*z* (Supplementary Fig. 14), which follows a similar exponential decay trend of the FDTD-simulated evanescent field generated at the second refraction position on the microsphere surface (Supplementary Fig. 15). The interaction of these evanescent waves and samples is considered to play a role in the origins of the sub-diffraction-limited resolution of the microsphere superlens, which is further supported by the consistency between the theoretically estimated resolution (*λ*/6.5–*λ*/9.2) by spectral analysis and the experimentally achieved values (*λ*/6.3–*λ*/8.4) (Supplementary Note 3). The apparent improvement in the partial and inclined illumination on imaging quality (Fig. 7d–m) and the range of Δ*z* that allows for acceptable imaging (Fig. 8c,d and Supplementary Fig. 4a,b) are supported by an analysis of the loss of deterioration from the middle illumination, which mainly introduces propagating light during imaging, and the transfer of the second refraction position by inclining illumination, which shortens the distance for evanescent waves transferring to the sample and induces a spectrum extension of the evanescent waves illuminated on the sample surface (Supplementary Note 3). The evanescent waves with high spatial frequencies generated by the microsphere are weaker than the propagating waves^42^ and the background, a situation that worsens as the frequency increases. This practical restriction prevents the microsphere superlens from achieving its theoretical resolution limit. The evanescent field can be enhanced by increasing the illumination intensity; however, this action is accompanied by a promotion of the propagating field. The proposed partial and inclined illumination provides an option to circumvent this problem (Supplementary Note 3).

## Discussion

We have shown that non-invasive super-resolution imaging over a large area can be achieved by properly developing the imaging properties of the microsphere superlens explored in this study. By replacing the detection point with a microsphere superlens, a non-invasive microsphere-based SSUM has been demonstrated, and the imaging acquisition efficiency has been improved by ∼ × 200 compared with a commercial AFM. Because the SSUM imaging time is primarily consumed by the scanning and image stitching processes, the imaging efficiency can be further improved by using more efficient algorithms for image stitching or using a higher-speed camera or parallel scanning techniques, such as parallel AFM^14^,^15^ or superlens arrays^43^,^44^,^45^. Structural information of the live-cell surface and sub-membrane nanostructures can be detected in a non-invasive mode with sub-diffraction-limited resolution. Although wet cells can be imaged at extremely high resolution via SEM under the protection of nanoscale thickness membranes^46^,^47^ or graphene^41^, considerable obstacles still exist for non-destructive cell imaging after more than six decades of development. There are also technical problems that must be solved to achieve simultaneous SEM and fluorescent imaging^48^. Conversely, the SSUM also enables super-resolution fluorescent imaging and is compatible with traditional fluorescent microscopes. Therefore, the proposed method has the potential to non-invasively realize live-cell structural information acquisition and specific molecule tracking concurrently over a large area, which facilitates studying rare subcellular events in their cellular environment and over a complete life period.

## Methods

### Microsphere-based SSUM system and imaging equipment

The BaTiO_3_ microspheres (supplied by Cospheric) were attached to the cantilever using an ultraviolet curable glue (NOA63, Edmund Optics) as shown in Fig. 1c,d. Different illumination conditions were achieved by adjusting the two stops in the Köhler illumination system (Thorlabs). A high-speed scientific complementary metal oxide semiconductor camera (PCO. Edge 5.5) was used to record the images. Illumination was provided by an intensity-controllable light source (C-HGFI, Nikon, Japan), and the peak illumination wavelength of the system was set to ∼550 nm by the optical components for white-light imaging. A × 50 objective (Nikon TU Plan EPI ELWD) and a × 100 objective (Nikon LU Plan EPI ELWD) were used in these experiments. A split photodiode (QP50-6-18U-TO8, First Sensor) and a 635 nm circular beam laser diode module (#83-838, Edmund Optics) were applied in the custom AFM system (Supplementary Fig. 1). Polystyrene (*M*_w_=123 kg mol^−1^, *M*_w_/*M*_n_=1.08, from Alfa Aesar) was dissolved in toluene and spin-coated onto the AgNWs that were dispersed onto silicone substrates. The optical beam profiler used to measure the illumination conditions was made by Thorlabs (BP209-VIS). The SEM images were taken using a Zeiss EVO MA10.

### Cell culture and imaging

Mouse myoblast cells (C2C12) and human breast cancer cells (MCF-7) were cultured on common Petri dishes in DMEM or RPMI-1640 containing 10% fetal bovine serum and 1% penicillin–streptomycin at 37 °C (5% CO_2_) in an incubator (Model 371, Thermo Scientific). The solvent used to fix the cells imaged in this study was 4% paraformaldehyde. For fluorescence microscopy of actin cytoskeleton, the cells were treated with Alexa Fluor 488-phalloidin (A12379, Thermo Fisher Scientific) at a concentration of 0.165 μM according to the process recommended by the supplier. A mercury lamp filtered by a fluorescein isothiocyanate (FITC) emission filter (MDF-FITC, Thorlabs) was used to excite fluorescence. The fluorescent imaging by SSUM (Fig. 6d and Supplementary Figs 7d and 8d) was conducted before the white-light imaging to prevent fluorescence quenching.

### Imaging with SSUM

The microsphere-attached AFM probe was mounted on a custom holder. After the common adjustment procedure used in AFM, the sample was moved towards the microsphere using a three-dimensional translation stage during which the AFM feedback was acquired to monitor whether the microsphere touched the sample surface and to adjust the distance between the microsphere and sample. As the distance or force reached a set-point value, the optical microscope was driven by a vertical translation stage with nano-scale resolution (IMS100V, Newport) to acquire the microsphere-generated virtual images. The AFM feedback was open or closed in contact or non-invasive mode. In non-invasive mode, Δ*z* was precisely adjusted by the PZT scanner (P-733.3CL, Physik Instrumente, Germany), and the sample stage was adjusted to be horizontal to decrease the difference in the distance between the microsphere apex and sample stage. The images were recorded by an external signal that triggered a high-speed camera from a controller (Supplementary Figs 1a and 16a) during scanning. The recorded area of the camera could be adjusted before scanning to the proper size to satisfy the overlap (15–20% in our experiments) required for image stitching and to be within the area of the FOV of the microsphere superlenses without apparent aberration, which effectively reduces the data processing time and enables rapid image processing during or after scanning. These recorded images could be directly used in the image stitching procedure without preprocessing. A commercial piece of software (Topostitch, Image Metrology) was used to stitch the acquired image tiles of a CPU, a Blu-ray disc, and the cell shown in Fig. 5b,d, whereas the free ImageJ software with the Stitching plugin^49^ based on a phase correlation algorithm^50^ was used to stitch the cellular images shown in Figs 5f,h and 6b,d according to the procedures shown in Supplementary Fig. 16c–i. These two software platforms took the recorded image tiles as input and the set overlap percentage. A few layout arrangements were provided for selection to arrange the image tiles in trigger-signal order (Supplementary Fig. 16a). Several fusion methods are also provided for selection by ImageJ. We mention both of them here to provide more options for different purposes or applications. There were 1,056 (Figs 2c, 4b and 5d,h), 561 (Fig. 6b,d), 272 (Fig. 3d) and 320 (Supplementary Fig. 5d) image tiles used in the image stitching processes. The performances of and time consumed by these two software platforms are compared in Supplementary Fig. 16 and Supplementary Table 1. The quality of the stitched images can be further improved by selective processing using a band-pass filter algorithm or a recursive bilateral filtering algorithm^51^.

### Analyses of microsphere imaging properties

In this study, the imaging conditions were similar to the constant-height scanning mode (that is, the microsphere was attached to a TESP cantilever, which was fixed to maintain the distance between the microsphere and objective, as shown in Fig. 7a). Initially, the microsphere superlens was in contact with the Blu-ray disc’s surface (that is, Δ*z*=0 μm) without pre-stress, which can be achieved by monitoring the position-sensitive device signal of the AFM system. Then, the microscope, including the objective driven by a motorized vertical stage, was adjusted to a position where the virtual images generated by the microsphere superlens could be clearly observed. Before scanning, a pre-stress was applied by moving the PZT towards to the objective; this produced an ∼1 μm-deep pre-deformation at the position, where the microsphere was attached. Then, the Blu-ray disc was carried by the PZT scanner away from the microsphere at different illumination conditions (Fig. 7b,c). The virtual images generated by microsphere superlens in the vertical scanning processes were recorded using a high-speed camera (inset of Fig. 7a).

### Data availability

The data sets generated during and/or analysed during the current study are available from the corresponding authors on reasonable request.

## Additional information

**How to cite this article:** Wang, F. *et al*. Scanning superlens microscopy for non-invasive large field-of-view visible light nanoscale imaging. *Nat. Commun.* **7,** 13748 doi: 10.1038/ncomms13748 (2016).

**Publisher’s note:** Springer Nature remains neutral with regard to jurisdictional claims in published maps and institutional affiliations.

## Supplementary Material

Supplementary Information Supplementary Figures, Supplementary Table, Supplementary Notes and Supplementary References.

Supplementary Movie 1 Dynamic scanning of a CPU surface by SSUM.

Supplementary Movie 2 Dynamic scanning of a skeletal muscle cell (C2C12) by SSUM.
