# Supplementary material for: Scanning superlens microscopy for non-invasive large field-of-view visible light nanoscale imaging
Source: Nat Commun. 2016 Dec 9;7:13748. doi: 10.1038/ncomms13748 (PMC5476830; doi:10.1038/ncomms13748)
Supplement: Supplementary Information — Supplementary Figures, Supplementary Table, Supplementary Notes and Supplementary References. [file ncomms13748-s1.pdf]

Supplementary Figures

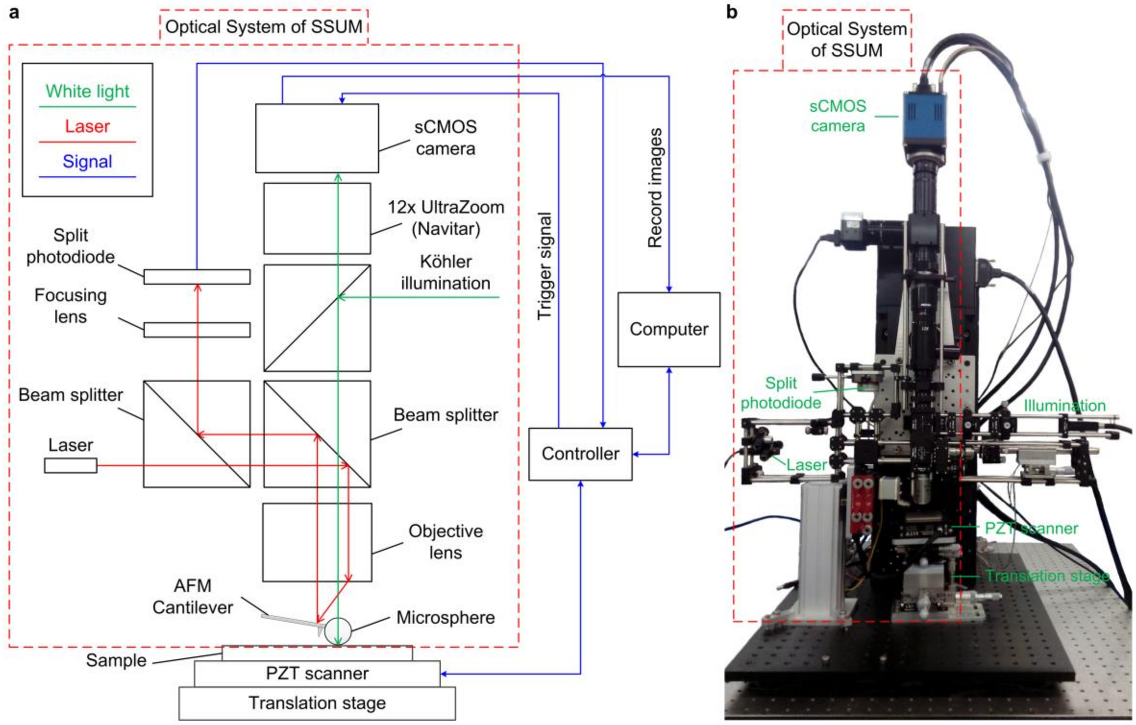

Supplementary Figure 1. Scanning Superlens Microscopy. (**a**) Schematic of the SSUM. (**b**) Photograph of the actual SSUM setup.

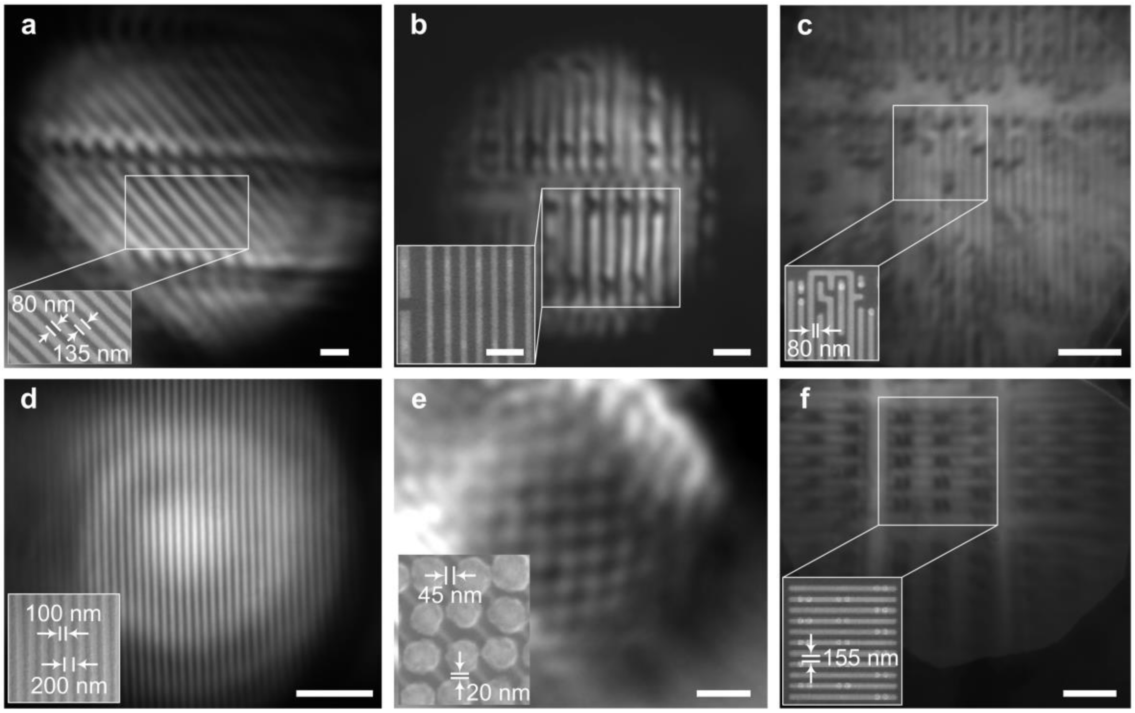

Supplementary Figure 2. Nanostructure imaging using the microsphere superlenses in water. Virtual imaging of the CPU surface using the $BaTiO_3$ microsphere superlenses with diameters of (**a**) 27 µm, (**b**) 33 µm, (**c**) 57 µm, (**e**) 19 µm and (**f**) 57 µm in water. (**d**) Image of a Blu-ray disk surface using a 70-µm-diameter $BaTiO_3$ microsphere in water. (**a**, **b**, **d**, **e**) A 50x (NA = 0.6) objective and (**c**, **f**) a 100x (NA = 0.8) objective were used. The magnifications due to the use of the microspheres were (**a**) 4.5x, (**b**) 4.1x, (**c**) 3.4x, (**d**) 3.2x, (**e**) 4.7x and (**f**) 3.4x. The scale bars shown in these images correspond to the actual size. The insets are SEM images. All of these observations were conducted under partial and inclined illumination. Scale bars: 500 nm (**a**); 1 µm (**b**); 2 µm (**c**,**f**); 3 µm (**d**); 600 nm (**e**).

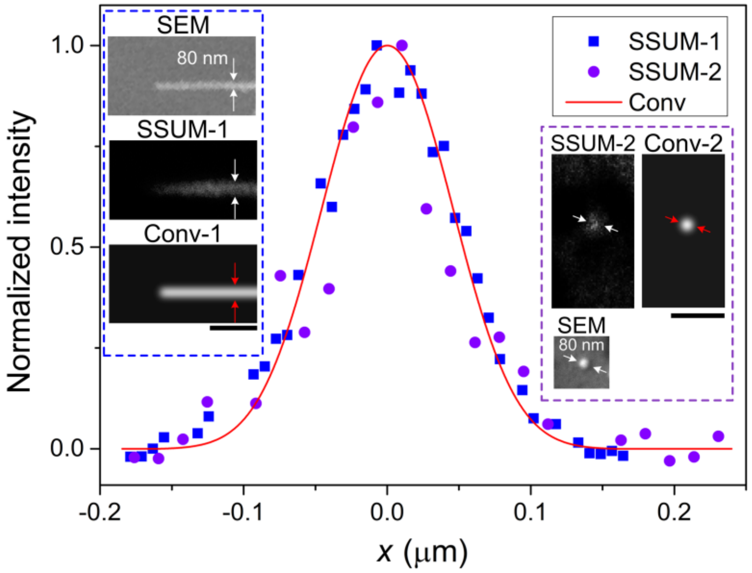

Supplementary Figure 3. Resolution calibration of SSUM. The insets are two sets of SEM images, SSUM and calculated convolution results. The scatter data (SSUM-1, SSUM-2) are used to estimate the resolution. The SSUM-1 is the cross section of a silver nanowire with a diameter of 80 nm and SSUM-2 is the cross section of an 80-nm diameter gold nanoparticle observed under partial and inclined illumination. The solid curve is a cross section of Conv-1 and Conv-2 representing the calibrated convolution result. Scale bars: 0.5 μm.

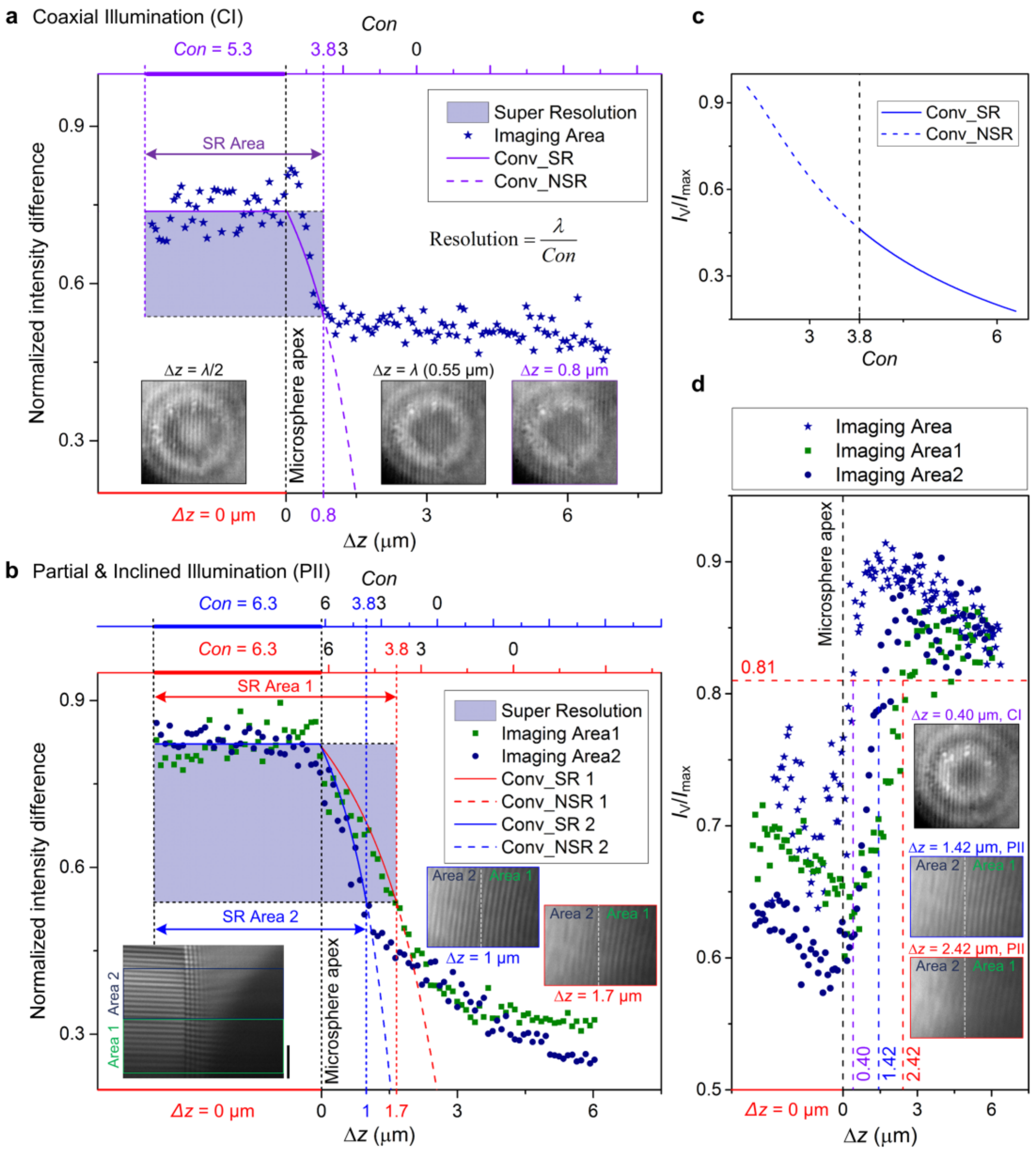

Supplementary Figure 4. Attenuation of resolution with Δ*z* at different illumination conditions. (**a**) Coaxial illumination. (**b**) Partial and inclined illumination. (**a**, **b**) Observation of a Blu-ray disk surface as shown in the SEM image in the inset of Supplementary Fig. 2d. Δ*z* is defined in Fig. 7a, normalized intensity difference (*NID*) and *Con* is defined in Fig. 8. The data used to calculate *NID* (scatter data) are extracted from the experimentally measured cross sections of the structures on the Blu-ray disc surface using a 60-μm diameter microsphere similar to that in Fig. 7d,e. The two scatter sets shown in (**b**) are calculated based on the data from the two areas marked in the inset. (**c**) The relationship between I_V/I_{max} calculated by the convolution results and *Con*. In (**a**, **b**, **c**), the solid curves representing super-resolution and dash curves standing for the diffraction confinement are achieved from the calibrated convolution results, e.g., the solid curve in Supplementary Fig. 3. (**d**) Comparison of I_V/I_{max} with the critical value (0.81) of the Rayleigh criterion. The incline angle, as defined in Fig. 7c, was set to ~ 70 ° for the experiments under partial and inclined illumination. Scale bars: 2 μm (**b**).

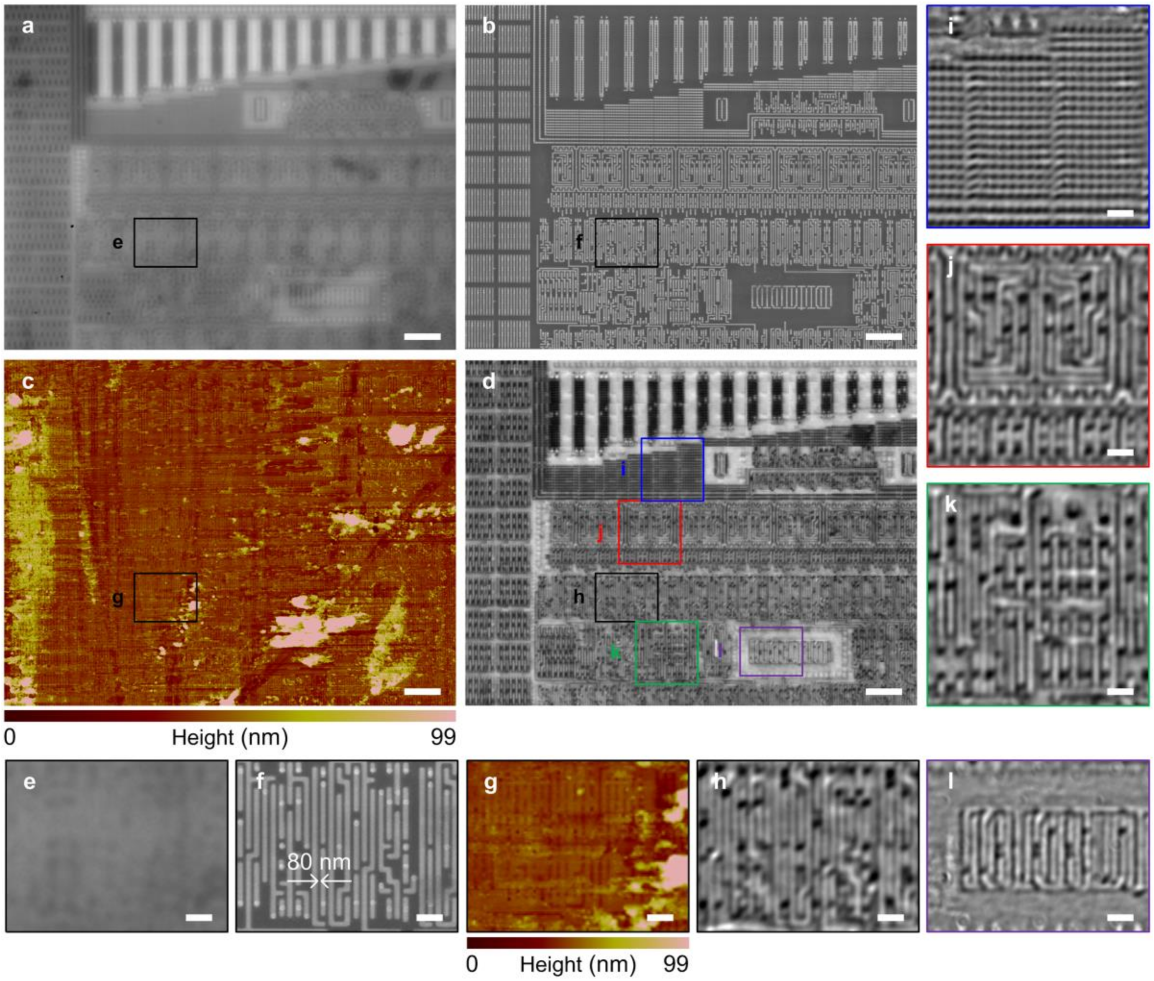

Supplementary Figure 5. CPU surface imaging in constant-height scanning mode. (**a**) The CPU surface directly observed using a 100x (NA = 0.8) objective; detailed structure information cannot be resolved. (**b**) SEM image. (**c**) AFM scanning image. (**d**) Large area imaged using the SSUM in constant-height scanning mode. A video recording of the scanning process is provided as Supplementary Video 1. The objective used in the microsphere scanning process is the same as in (**a**). (**e-l**) are local magnified areas that correspond to the marked areas in (**a-d**). Scale bars: 5 μm (**a-d**); 1 μm (**e-l**).

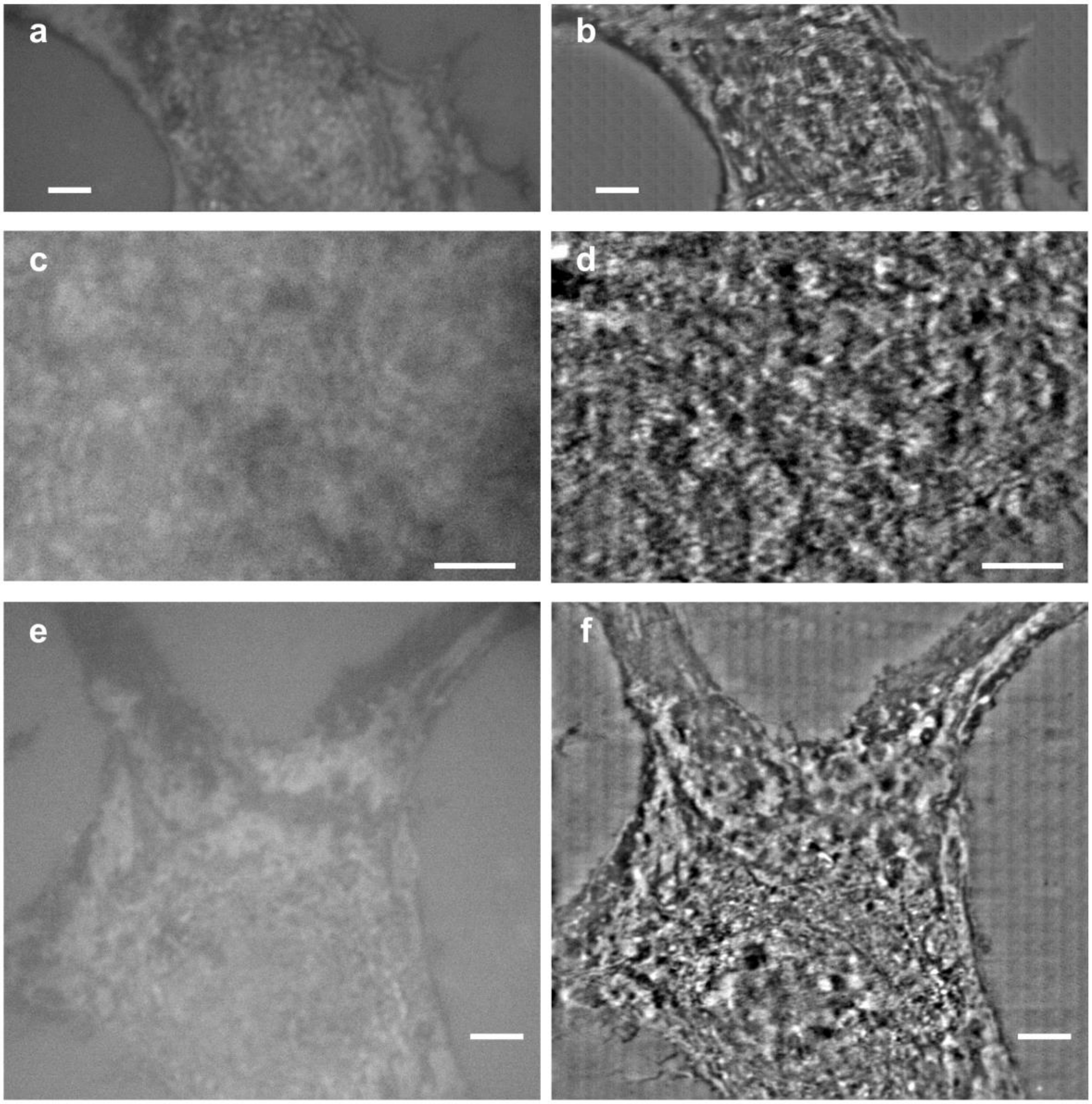

Supplementary Figure 6. Additional SSUM images of the C2C12 cells. C2C12 cells were observed (**a**, **c**, **e**) without and (**b**, **d**, **f**) with the aid of the microsphere superlens. A 100x (NA = 0.8) objective was used in these experiments. Scale bars: 5 μm (**a**,**b**); 6 μm (**c**-**f**).

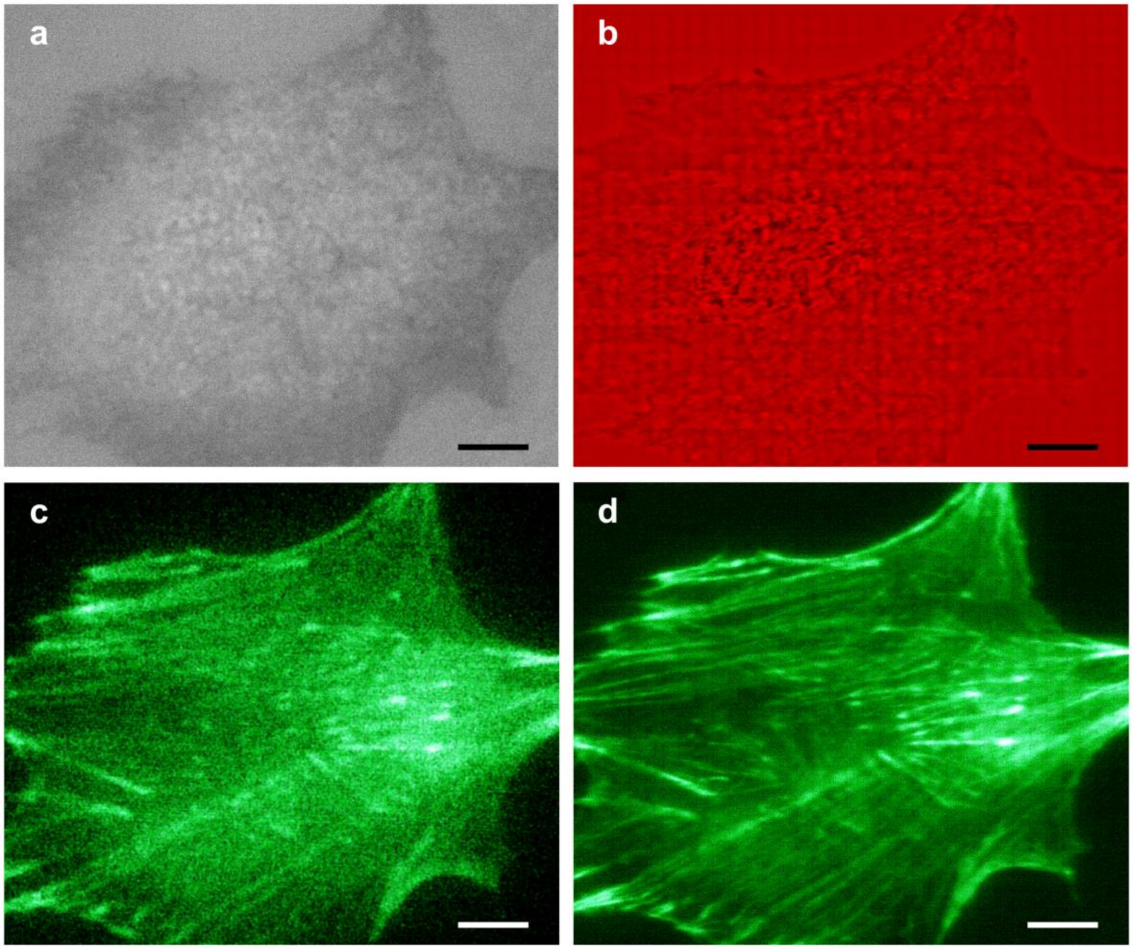

Supplementary Figure 7. Additional SSUM images for comparison of white-light and fluorescent imaging. A C2C12 cell observed (**a**, **c**) without and (**b**, **d**) with the enhancement provided by the microsphere superlens. (**a**, **b**) White-light images. (**c**, **d**) Fluorescent images. A 100x (NA = 0.8) objective was used. Scale bars: 8 μm.

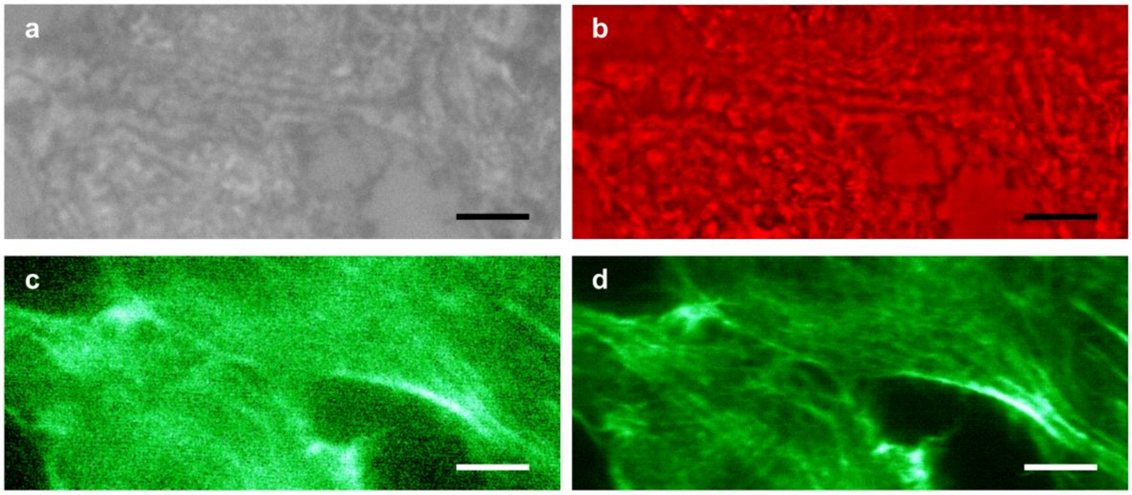

Supplementary Figure 8. Additional SSUM images for comparing white-light and fluorescent imaging. A C2C12 cell imaged (**a**, **c**) without and (**b**, **d**) with the enhancement provided by the microsphere superlens. (**a**, **b**) White-light images. (**c**, **d**) Fluorescent images. A 100x (NA = 0.8) objective was used. Scale bars: 8 μm.

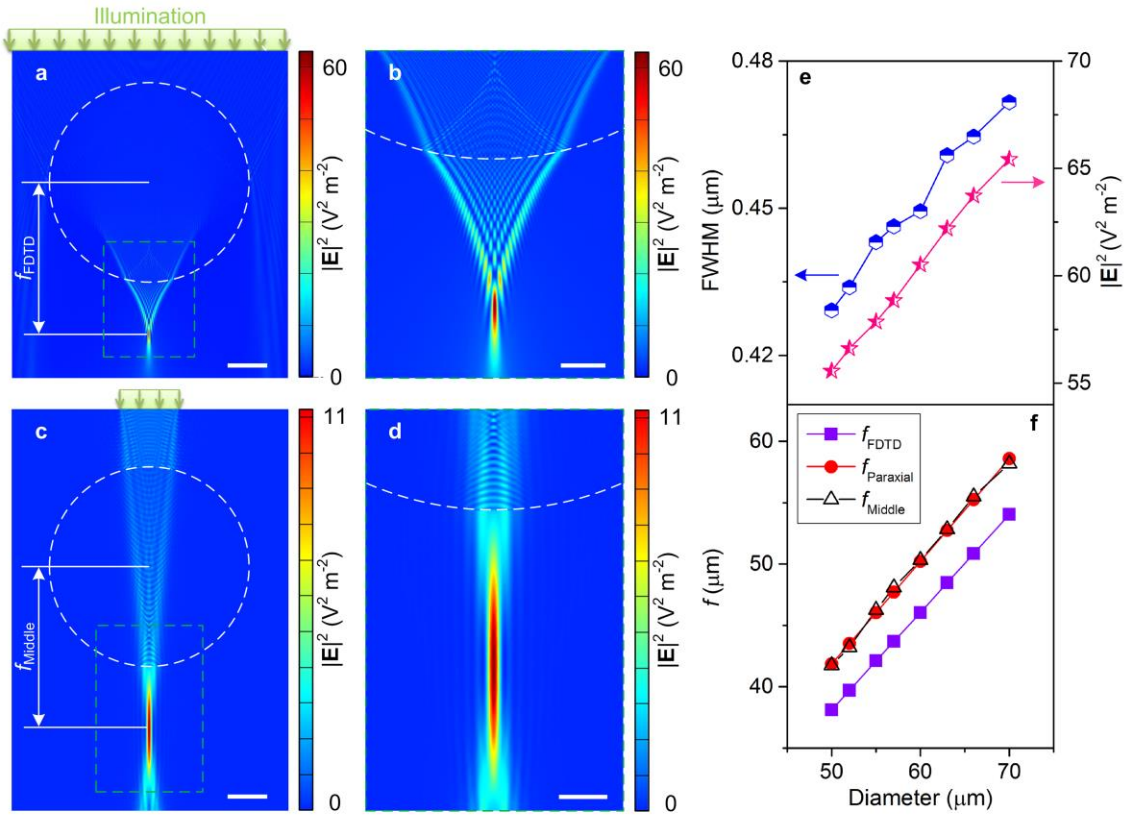

Supplementary Figure 9. Focusing characteristics of the microsphere superlens. (**a-d**) Simulated electric field intensity distribution around the microsphere superlenses under different illumination conditions based on the FDTD computational technique. (**e**, **f**) show the relationship between the focus full-width at half-maximum (FWHM), the intensity ($|\mathbf{E}|^2$) of focus spot, the focal length (f_{FDTD}) under illumination covering the microsphere, the focal length (f_{Middle}) under illumination localized in the middle area of the microsphere superlens, paraxially calculated focal length (f_{Paraxial}) and microsphere diameter in the range of 50 to 70 μm. Scale bars: 10 μm (**a**,**c**); 5 μm (**b**,**d**).

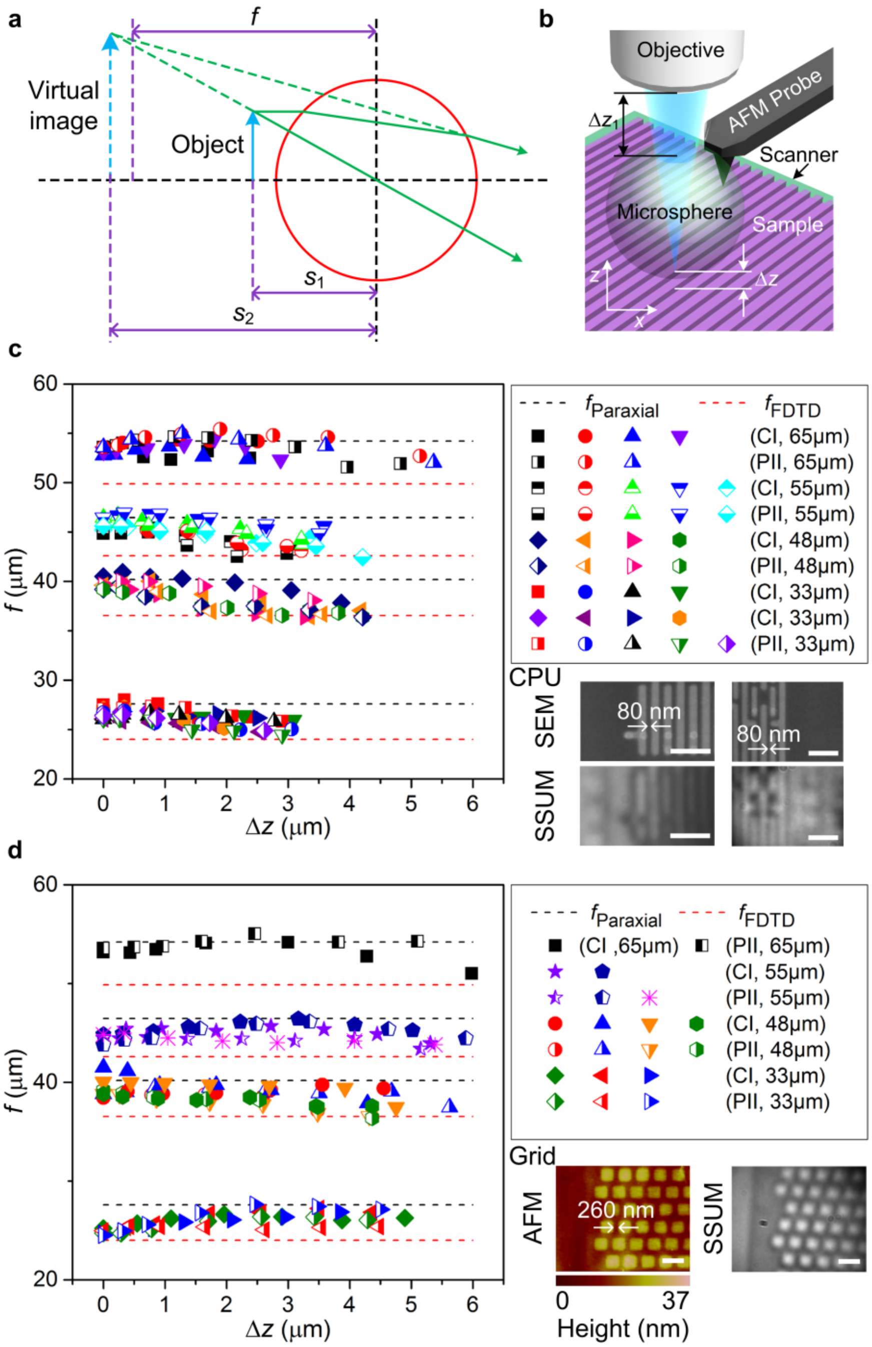

Supplementary Figure 10. Experimental study of microspheres' focal length. (**a**) Schematic showing virtual image formation of a sphere by ray tracing. (**b**) Schematic for the experimental setup used in this study. Experimentally calibrated focal lengths (f) of microsphere superlenses with diameters of 65 μm, 55 μm, 48 μm and 33 μm based on the measured object distance (s_1) and corresponding image distance (s_2) during observing structures with (**c**) sub-diffraction-limited features and (**d**) those larger than the diffraction limit under coaxial illumination (CI) or partial and inclined illumination (PII), as shown in Fig. 7. The inset SEM, AFM and SSUM images in (**c**, **d**) show the structures used in these experiments, respectively. Scale bars, 1 μm (**c**, **d**).

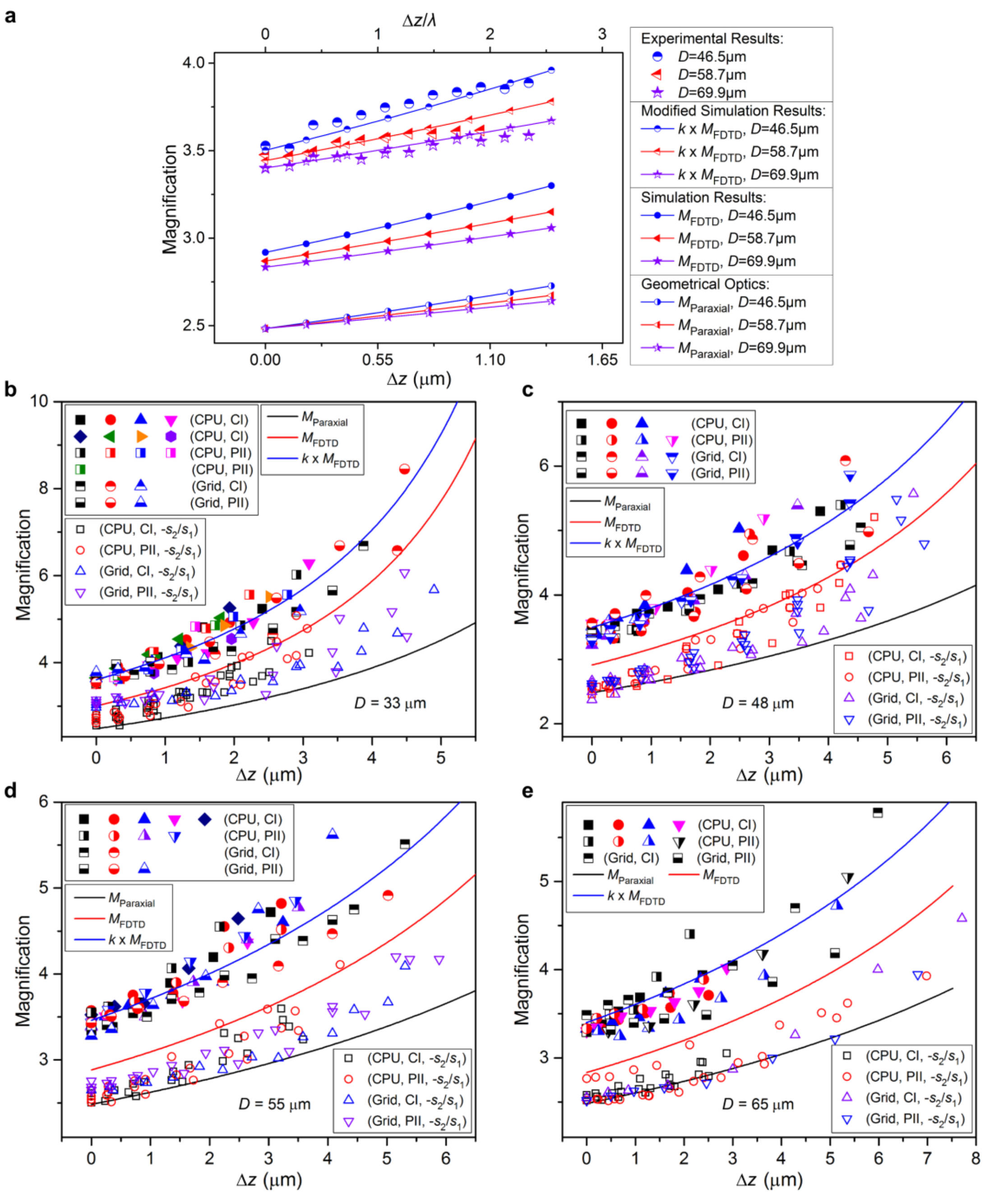

Supplementary Figure 11. The virtual image magnification factors of microsphere superlens. (**a**) Experimental results were obtained by analyzing the virtual images recorded during PZT scanning for different diameters as Δz_1 is adjusted for the clearest imaging when Δz = 0 μm and Δz_1 does not change during PZT scanning (Supplementary Fig. 10b). The simulation results were calculated by the FDTD method. Geometrical optics results were computed by paraxial functions. (**b-e**) The solid and half solid scatter points are the magnification factors obtained by analyzing the recorded virtual images at different Δz values while Δz_1 is adjusted for the clearest imaging. The hollow points are calculated by $M = -s_2/s_1$. The CPU and grid samples are the same as Supplementary Fig 10c,d.

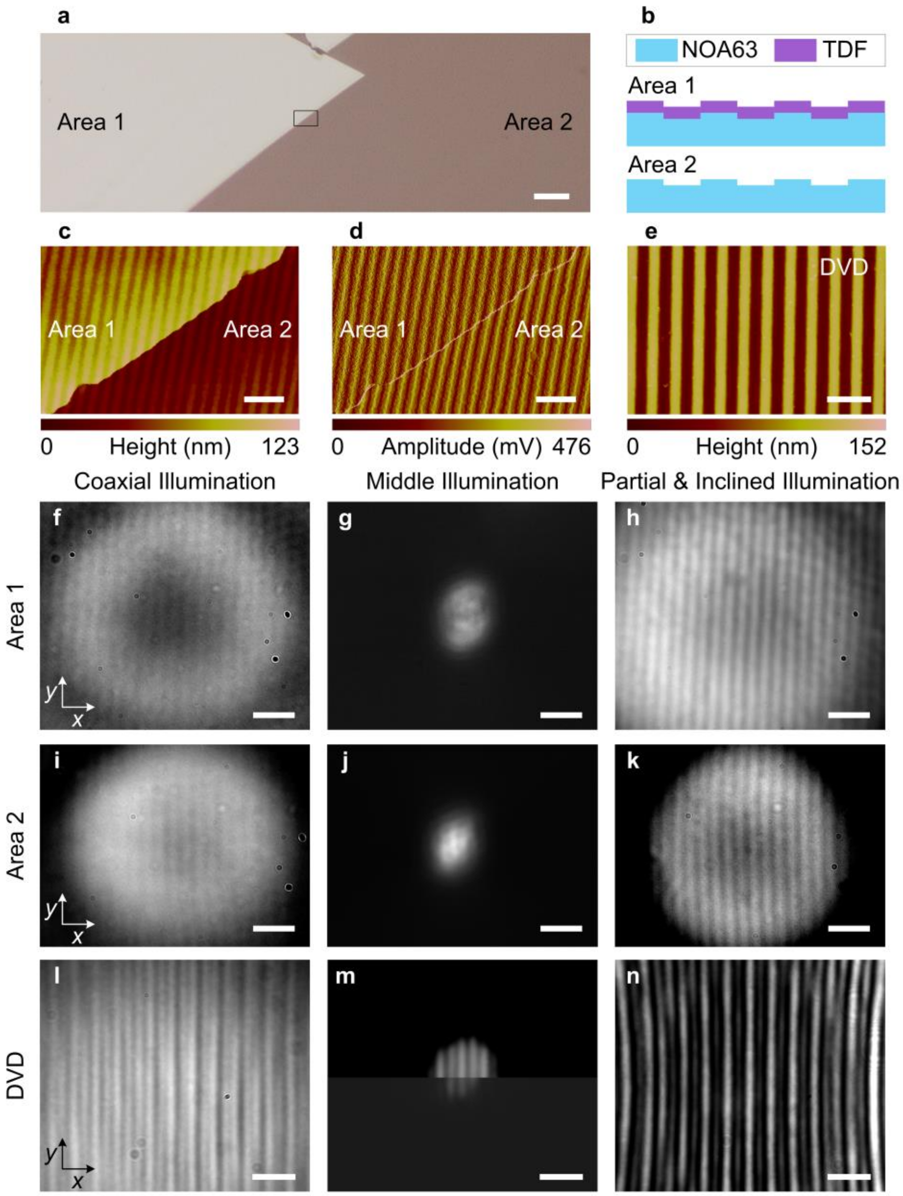

Supplementary Figure 12. Observation of transparent/ low-reflectivity samples by SSUM. The transparent samples were achieved by separating the photo-cured transparent NOA63, which was dripped onto a Blu-ray disc surface with ~ 100 µm of the transparent protection film removed from the Blu-ray disc. (**a**) Two imaging areas were observed with an optical microscope mounted with a 0.5-NA objective. In Area 1, some transparent dielectric films (TDF) on Blu-ray disc surface with thickness of ~ 55 nm are left on the NOA63 substrate. Area 2 is the NOA substrate with sub-diffraction-limited structures copied from the Blu-ray disc. (**b**) A schematic of the surface, AFM scanned (**c**) height and (**d**) amplitude images of these two areas. (**e**) AFM image of structures on the digital versatile disc (DVD) surface. Structures on (**f-h**) Area 1, (**i-k**) Area 2 and (**l-n**) the DVD surface were observed by microsphere superlenses under different illumination conditions. (**m**) The width of the illumination beam used by the upper image is larger than that used in the lower image. Scale bars, 10 µm (**a**); 1 µm (**c**, **d**, **f-k**); 1.5 µm (**e**, **l-n**).

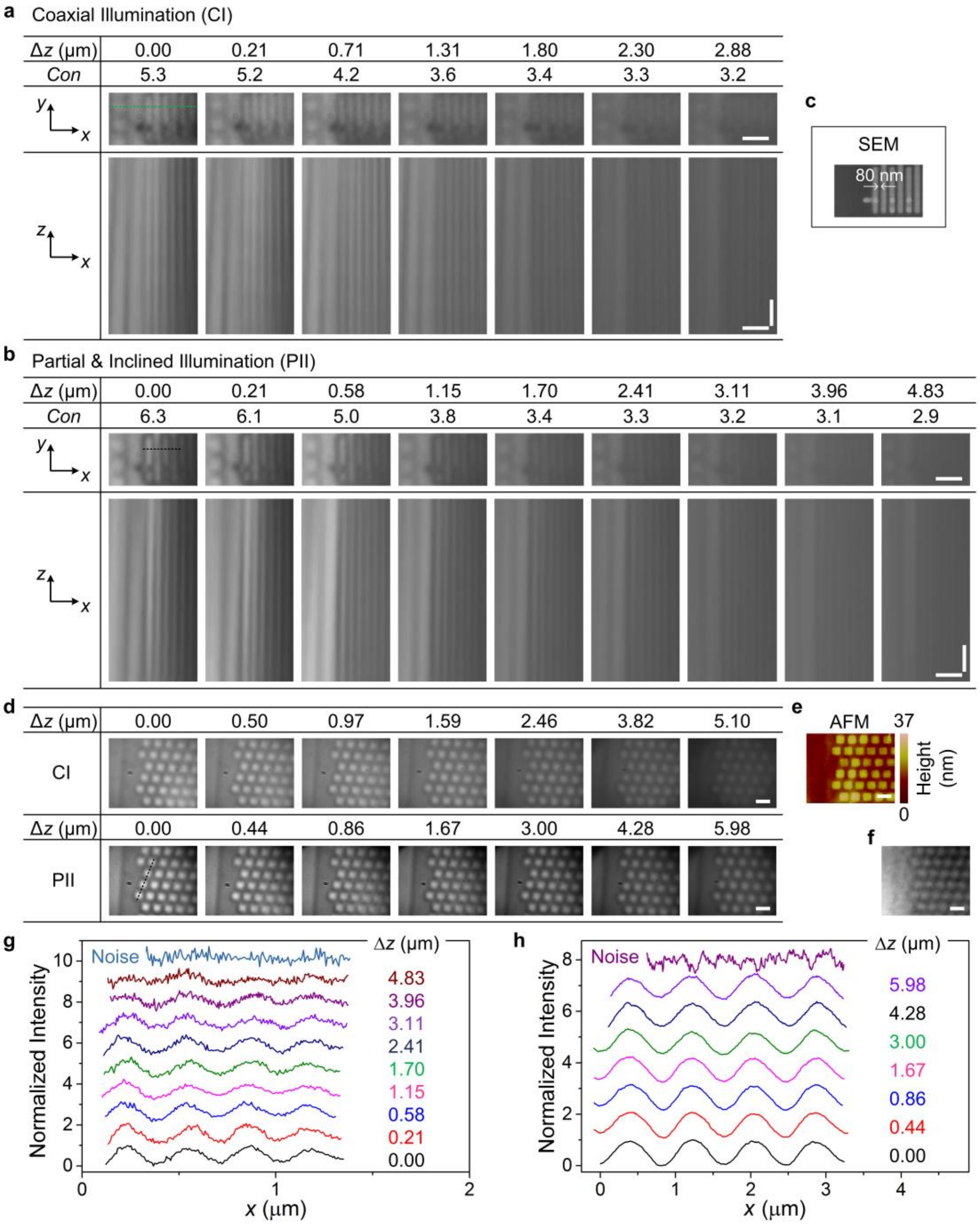

Supplementary Figure 13. The influence of Δ*z* on the observation of structures smaller or larger than the diffraction limit. (**a-b**) Sub-diffraction-limited structures (see SEM image in (**c**)) in CPU observed under (**a**) coaxial illumination and (**b**) partial and inclined illumination at different Δz values by SSUM mounted with a 65-μm diameter microsphere. (**d**) Observation of a grid with structures larger than the diffraction limit at different Δz values. (**e**) AFM image and (**f**) an image recorded by an optical microscopy mounted with a 0.8-NA objective of the structure observed in (**d**). (**g**, **h**) Normalized intensity of the profile marked by the dotted lines in (**b**, **d**), respectively. The noise signals are the normalized intensity of randomly selected profiles in smooth areas. Scale bars, 1 μm (**a**, **b-f**).

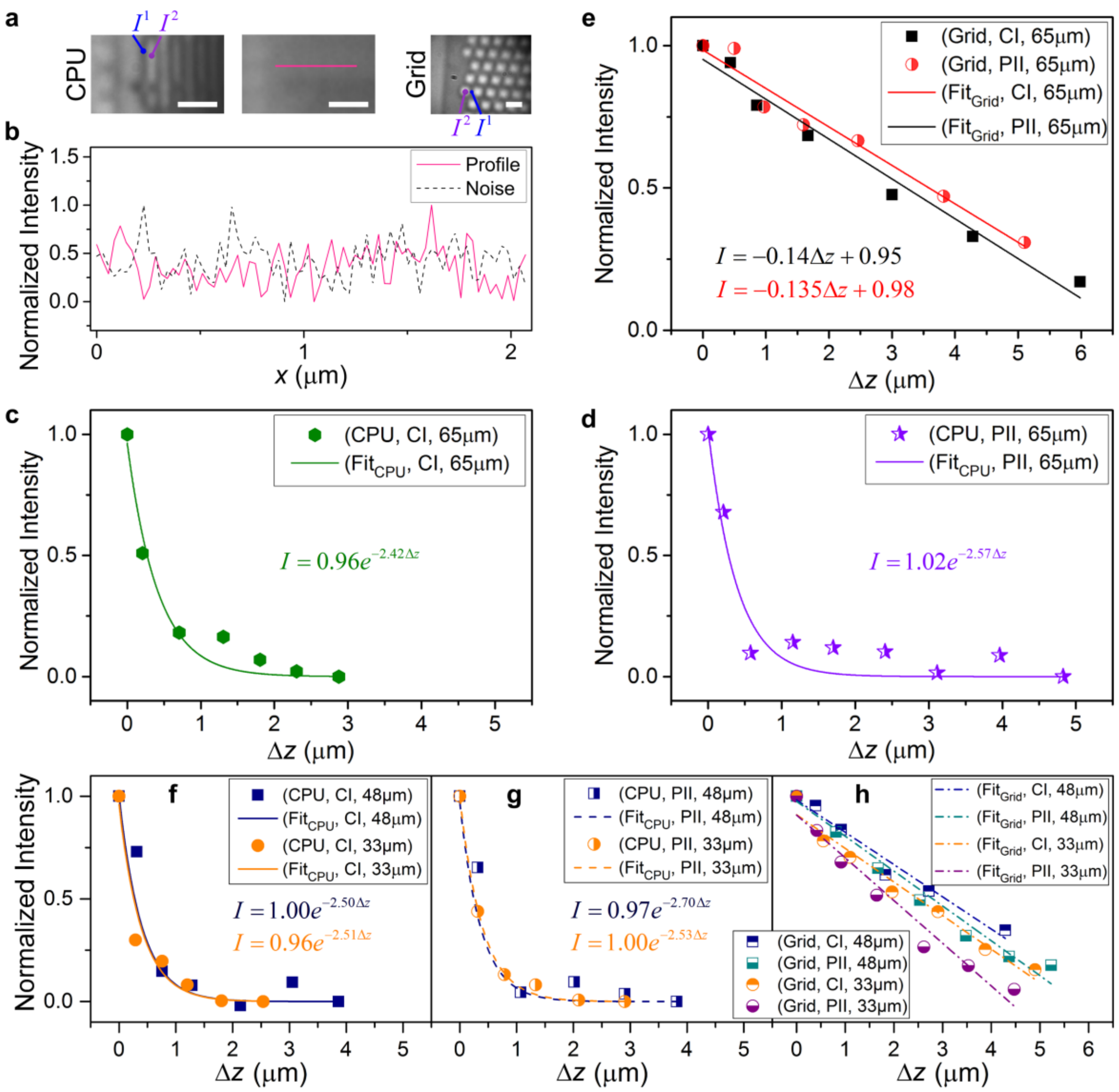

Supplementary Figure 14. The difference of intensity attenuation induced by the separation between the microsphere and samples containing features smaller or larger than the optical diffraction limit. (**a**) CPU structures observed by (left) SSUM, which correspond to the results shown in Supplementary Fig. 13b, or (middle) an optical microscope mounted with a 0.8-NA objective without the help of the microsphere. Right: A grid resolved by SSUM corresponding to the image shown in Supplementary Fig. 13d. (**b**) The solid curve is the normalized intensity along the line marked in the middle image of (**a**). The noise is the normalized intensity of a randomly selected profile in a smooth area. (**c-h**) Intensity attenuation induced by Δ*z* when structures (**c**, **d**, **f**, **g**) smaller or (**e**, **h**) larger than the optical diffraction limit are resolved by microsphere superlenses with diameters of 65 μm, 48 μm and 33 μm under different illumination conditions. The data used to calculate the results shown in (**c-e**) are extracted from Supplementary Fig. 13a,b,d, respectively. Scale bars, 1 μm (**a**).

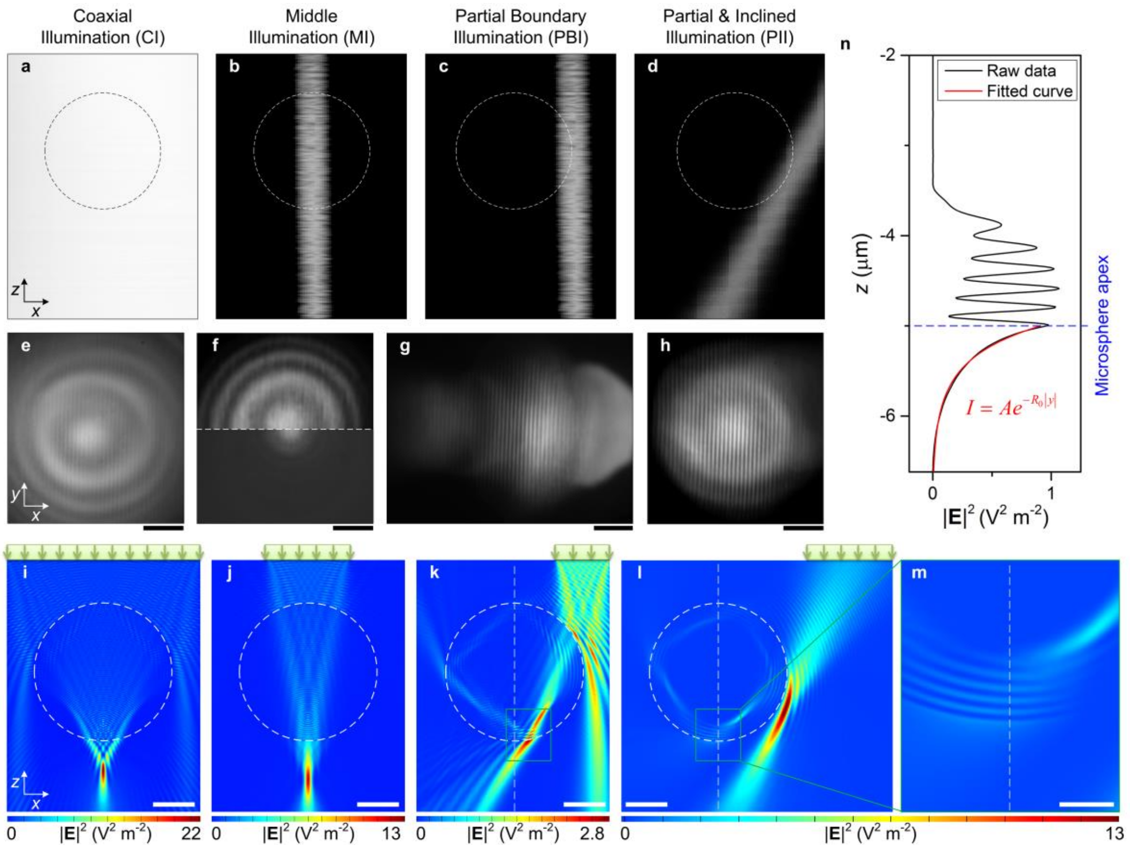

Supplementary Figure 15. Adjustments of illumination condition-induced enhancement of the microsphere-based imaging. (**a-d**) Four different illumination conditions detected by vertically scanning an optical beam profiler around the objective focus. (**e-h**) A Blu-ray disc surface was observed under different illumination conditions corresponding to each case shown in (**a-d**). (**f**) The width of the illumination beam used by the upper image is larger than that used in the lower image. The SEM image of the Blu-ray surface is shown in the inset of Supplementary Fig. 2(d). (**i-l**) The FDTD-simulated electric field intensity distribution around the microspheres under different illumination conditions corresponding to each case shown in (**a-d**). (**m**) Local magnified area of the marked area shown in (**l**). (**n**) Electric field intensity distribution along the vertical line shown in (**m**). The red curve represents the exponentially fitted results. A = 970577, R_0 = 2.775. Scale bars, 3 μm (**e-l**); 1 μm (**m**).

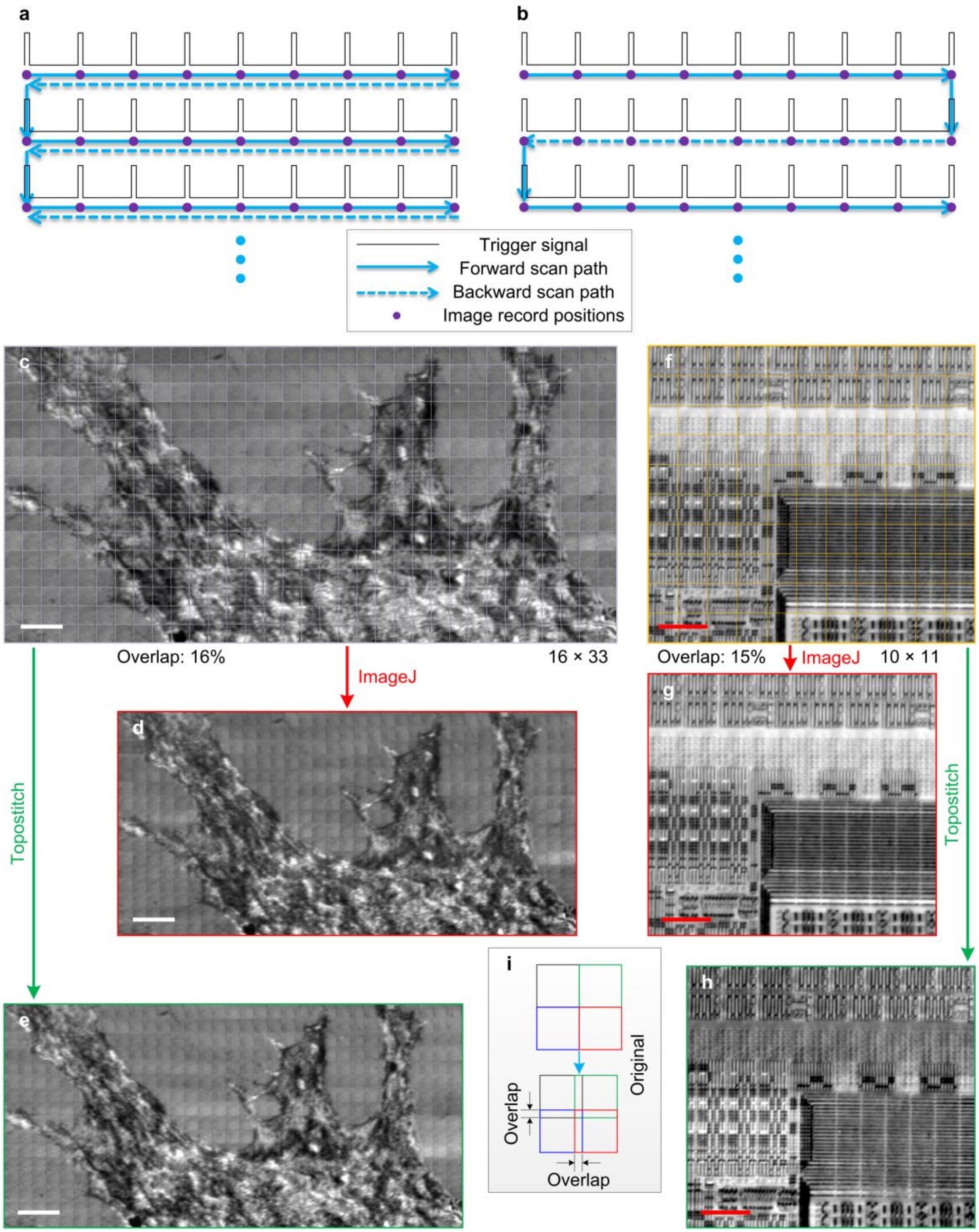

Supplementary Figure 16. Stitching image tiles recorded by SSUM. (**a**, **b**) Two possible scanning modes. Only scanning mode (**a**) is used in our experiments due to the equipment confinement. The proposed mode (**b**) can further increase the imaging efficiency by two-fold. (**c**, **f**) Original image tiles of a C2C12 cell and the structure of a CPU recorded during scanning microsphere superlens with diameters of 59 μm and 48 μm. Image stitching using (**d**, **g**) ImageJ and (**e**, **h**) Topostitch. (**i**) Schematic shows the original image tiles with overlapping regions corresponding to (**c**,**f**). Scale bars, 6 μm (**c**-**h**).

Supplementary Tables

Supplementary Table 1: Comparison of stitching time consumed by different software packages under different conditions

Software / Tile Num. / Pixel Size / Time (s)[b] / Mode[a]	ImageJ		Topostitch	
Tile Num.	272 (16x17)	1056 (32x33)	272 (16x17)	1056 (32x33)
Pixel Size	166 x 166			
x	-	-	3.2	10.7
y	-	-	3.4	12.6
x, y	65.1	283.5	3.5	13.2
Non	9.3	32.9	2.5	9.0

Tile Num. = The number of image tiles used in stitching.

[a]There are two stitching modes provided by ImageJ and four modes by Topostitch, which are denoted by "x", "y", "x, y" and "Non", respectively, i.e., the position of each tile is adjusted only along the x-axis, only along the y-axis, for both the x- and y-axes, or not translated. After this image placement, the tiles are fused together by a specific algorithm.

[b]Computer information: Windows 7 (64 bit)/ 8 GB RAM/Intel(R) Core(TM) i7-4790 CPU.

Supplementary Notes

Supplementary Note 1 - Definition of optical super-resolution and resolution calibration of SSUM

In the case of an imaging system with numerical aperture, NA = $n_0 \times \sin\theta$, two point sources with the same intensity can be resolved as a distance larger than $K\lambda$/NA, where n_0 is the object-space refractive index, θ is the half acceptance angle of objective, λ is the illumination wavelength, and K = 0.473, 0.5, 0.515 or 0.61 corresponding to the Sparrow[1], Abbe[2], Houston[3], or Rayleigh[4] criteria, respectively. As the microsphere contacts the sample, the solid-immersion concept is considered to estimate the resolution of microsphere-based diffraction-limited imaging as ~ $\lambda/2n_s$, where n_s is the refractive index of the microsphere[5,6]. When n_s = 1.9 (barium titanate glass microspheres), $\lambda/2n_s = \lambda/3.8$. The microsphere-based optical super-resolution is defined as the resolution higher than $\lambda/3.8$.

In addition to the resolution calibration in the main text, we also quantified the resolution of SSUM by 80-nm diameter silver nanowires and gold nanoparticles based on the process of convolution with a two-dimensional point-spread function (PSF)[5-7]. By matching the calculated convolution results to the experimental profiles, the resolution of SSUM mounted with a 57-μm microsphere is calibrated as FWHM = $\lambda/6.3$ (Supplementary Fig. 3), which is consistent with the results shown in Fig. 8a. Based on the analyses of Allen *et al.*, structures do not need to be resolved with extremely small features to calibrate the resolution[5]. For the case of white-light SSUM imaging, the peak illumination wavelength of illumination was set to 550 nm by the optical components. The resolution is calculated as FWHM = $\lambda/6.3 \approx 87$ nm. As the microsphere diameter decreases to 27 μm (Supplementary Fig. 2a), the resolution is estimated as $\lambda/8.4 \approx 65$ nm under partial and inclined illumination, which is consistent with the previously reported conclusion that smaller microspheres exhibit higher resolutions[8]. These results indicate that the resolution of SSUM is ~ $\lambda/6 - \lambda/8$ as a 30 – 60 μm microsphere used under partial and inclined illumination conditions.

We also studied the influence of Δz on the resolution of microsphere superlenses by observing the structures on a Blu-ray disc surface, on which the distance between microspheres and objective does not change (Supplementary Fig. 4a, b). Because the structures on the Blu-ray disc surface are neatly arranged lines, as shown in the inset of Supplementary Fig. 2d, no I_{min} can be acquired. However, the I_{min} can be estimated from the resolution calculated from the CPU results (Fig. 8c,d) considering that the resolution of a microsphere superlens does not change as Δz = 0 μm. *Con* = 3.8 is the critical value for the definition of optical super-resolution of microsphere-based imaging. Comparing the scatter data and curves, the resolution of the microsphere superlens is attenuated as the sample becomes farther from the microsphere apex from $\lambda/5.3$ or $\lambda/6.3$ to $\lambda/3.8$ for

different illumination conditions (see Fig. 8c,d and Supplementary Fig. 4a,b). The maximum separation of microsphere and sample enabling super-resolution capability of a 60-µm microsphere is approximately 0.65 – 0.8 µm for coaxial illumination and 1.0 – 1.7 µm for partial and inclined illumination (Fig. 8c,d and Supplementary Fig. 4a,b). In contrast, the Rayleigh criterion indicates that two one-dimensional objects can be distinguished, as the ratio between the (minimal) intensity of the central dip and the maximal intensity is smaller than 0.81 [9-11], i.e., $I_V/I_{max} < 0.81$. The influence of Δz on I_V/I_{max} for different illumination conditions is shown in Supplementary Fig. 4d. For the coaxial illumination and structures on the Blu-ray disc surface, the critical value (Δz = 0.40 µm) estimated by I_V/I_{max} is smaller than the *NID* estimated (Δz = 0.80 µm). However, as $0.40 \leq \Delta z < 0.8$ µm, the structures on the sample can still be clearly resolved (see insets in Supplementary Fig. 4a), and the microsphere still contains super-resolution capability (*Con* > 3.8) based on the resolution calibration process, which is inconsistent with the Rayleigh criterion. Under partial and inclined illumination, the critical values (Δz = 1.42 or 2.42 µm) estimated by I_V/I_{max} are larger than the *NID* obtained (Δz = 1.00 or 1.70 µm) for different areas. The images at the critical values of Δz estimated by I_V/I_{max} are blurry, which may reach the resolvable limit of the Rayleigh criterion. In the calculated I_V/I_{max} range, the corresponding *Con* is in the diffraction confinement range even at Δz = 0 µm (the sample contacts with the microsphere), comparing the results shown in Supplementary Fig. 4c,d, which is in contrast with previously reported results regarding the sub-diffraction-limited resolution capability of microsphere superlenses[5-8,12]. One possible reason is that the criteria based on I_V/I_{max} is sensitive to measurement and subsequent image processing procedures[11], such as the adjustment of image brightness and contrast during image record. Therefore, the *NID*-based method yields a better estimation for both cases considering image quality and resolution.

Supplementary Note 2 – Magnification factor of microsphere superlens-generated virtual images

The dependence of the virtual image magnification factor of the microsphere superlens on the distance (Δz) between the microsphere apex and samples is studied based on the comparison of an approximation of geometrical optics and finite-difference time-domain (FDTD)-simulated results with experimental results. The FDTD-based simulations were performed at the peak illumination wavelength (λ = 550 nm) and under perfectly matched layer boundary conditions (Lumerical FDTD Solutions). The paraxial approximation of sphere's focal length is $f_{\text{Paraxial}} \approx nD/4(n-1)$, where D is the microsphere diameter and n is the ratio of the refractive index of the microsphere to that of the medium (water). The FDTD results are smaller than the paraxial focal lengths (see Supplementary Fig. 9) due to the consideration of evanescent waves[13]. The paraxial conditions can be simulated as the illumination confined to the paraxial area, e.g., by limiting the illumination width to

15 μm, as illustrated in Supplementary Fig. 9c,d,f. Virtual images are observed in the experiments because the samples are set between the microsphere apex and focus and the optical microscope is focused below the sample surface. We further experimentally examine the focal length of microsphere superlenses with diameters of 65 μm, 55 μm, 48 μm and 33 μm, as shown in Supplementary Fig. 10. For a certain value of Δz, we can achieve a position where the clearest virtual image is observed through separately controlling Δz using a PZT stage and the distance (Δz_1) between the microsphere and the objective of microscope using a translation stage (M-IMS100V, Newport), as shown in Supplementary Fig. 10b. Using the experimentally measured position information, we can obtain the object distance (s_1) and the corresponding image distance (s_2). With this information, we can calculate the focal length using the Gaussian Lens Formula ($1/f = 1/s_1 + 1/s_2$),[14] where s_1 and s_2 are shown in Supplementary Fig. 10a. Repeated experimental results recording the observation of structures larger (semiconductor grid chip) or smaller (CPU) than the diffraction limit through microsphere superlenses with different diameters under different illumination conditions are used to calculate the focal length (Supplementary Fig. 10c,d). These calculated focal lengths based on the experimental data are bounded by the approximation of geometrical optics and FDTD-simulated results. Additionally, the observed sample size does not influence the calculated focal length compared to the results shown in Supplementary Fig. 10c,d.

Based on the schematic shown in Fig. 7a, the virtual image magnification of a sphere is given by $M_{\mathrm{Paraxial}} \approx f_{\mathrm{Paraxial}}/(f_{\mathrm{Paraxial}} - (D/2+\Delta z))$. When the distance between the sample and microsphere is considerably smaller than the microsphere radius ($\Delta z << D/2$), the magnification is simplified as $M_{\mathrm{Paraxial}} \approx n/(2 - n)$. We have also calculated the magnification factors (M_{FDTD}) by replacing the paraxial focal length with the FDTD-simulated focal lengths (f_{FDTD}). Supplementary Figure 11 shows the relationship between the magnification factors and Δz. We conducted experiments under two different conditions: (1) the distance (Δz_1 in Supplementary Fig. 10b) between the microsphere and objective is adjusted to a constant value for which the clearest imaging can be observed when $\Delta z = 0$ μm; i.e., Δz_1 does not change as Δz is adjusted by the PZT scanner. This condition is studied because the fixed Δz_1 is an experimental condition of SSUM for large-area imaging. (2) For a certain Δz, Δz_1 is adjusted to achieve the clearest image. A Blu-ray disc (Supplementary Fig. 11a), a CPU with sub-diffraction-limited structures and a grid containing features larger than the diffraction limit (Supplementary Fig. 11b-e) are observed. The actual virtual image magnification factors of microsphere superlenses with different diameters under the first (Supplementary Fig. 11a) or second (Supplementary Fig. 11b-e) experimental condition can be obtained by analyzing the recorded images at different Δz values (solid or half solid scatter points in Supplementary Fig. 11). Furthermore, we also calculate the magnification based on the experimentally measured object and image distances as $M = - s_2/s_1$ (the hollow scatter points shown in Supplementary Fig. 11b-e) [14]. These calculated magnification factors ($M = - s_2/s_1$) are

bounded by the approximation of geometrical optics and FDTD-simulated results but deviate from the results (the solid or half solid scatter points shown in Supplementary Fig. 11b-e) directly measured from the recorded images. The observed phenomena are not influenced by the sample size, whether larger or smaller than the diffraction limit. In addition, the virtual image magnification factor changes with the microsphere diameter when the microsphere is in contact with samples (Δz = 0 μm)[8], as shown in Supplementary Fig. 11, which deviates from the approximation of geometrical optics ($n/(2 - n)$) that changes only with the refractive index. Therefore, the approximation of geometrical optics is unable to describe the magnification property of the microsphere superlens. Wang *et al*. presented a fitting formula to describe the near-field magnification factor of microspheres on a sample surface[15]. However, the description of the relationship between the near-field magnification with Δz is still not reported in the literature, though the relationship between the magnification with Δz in the far field has been presented in the limit of geometrical optics as M_{Paraxial} [5]. Here, we present another fitting formula based on the magnification factor calculated by the FDTD-simulated focal lengths, $M \approx k \times f_{\text{FDTD}}/(f_{\text{FDTD}} - (D/2 + \Delta z))$. The factor $k \approx 1.2$ is found to correspond well with the experimental results obtained under different experimental conditions (Supplementary Fig. 11).

Supplementary Note 3 - Exploring the imaging mechanism and the enhancement of microsphere-based imaging by adjusting the illumination conditions

A debate regarding the fundamental imaging principle of "super-resolution" has been underway since the super-resolution capability of specific microspheres was first demonstrated in 2011 by Wang *et al.*[15]. Several mechanisms have been proposed to explain the enhanced resolution phenomenon of the microsphere superlens, such as the function of super-resolution foci[15], optical resonance effects[16], the enhancement of evanescent fields[17], resonant plasmon[18] and coherent effects[19], among other lines of reasoning[7,20,21]. The observed high resolution is difficult to sufficiently explain by the photonic nanojets owing to the limited FWHM of $\lambda/3 - \lambda/2$.[22,23] Here, we analyze the imaging enhancement by adjusting the illumination conditions and imaging mechanism by comparing experimentally acquired and numerically calculated results, as shown in Supplementary Figs. 12 – 15.

To exclude the influence of the samples' reflectivity on the observed results and following analyses, we conducted experiments on samples with different reflectivities under different illumination conditions. Supplementary Fig. 12f-k and Supplementary Fig. 15e-h show images of a transparent sample consisting of structures copied/transferred from the Blu-ray disc surface and a Blu-ray disc embedded with a reflective layer, respectively. Figure 8 and Supplementary Figure 13a,b,d also illustrate the observation of

structures in the CPU and semiconductor grid chip, respectively, under different illumination conditions. All of these results are consistent; i.e., the sample reflectivity has a negligible effect on the analyses.

We start our discussion with the experimental study of the influence of Δz on the imaging quality of microsphere superlenses under the second experimental condition described in the Supplementary Note 2; i.e., for a certain Δz, Δz_1 is adjusted to achieve the clearest imaging (Supplementary Fig. 13). The images (*x*-*y*) in the third row of Supplementary Fig. 13a,b are the clearest images obtained at different Δz values by scanning a translation stage on which the microscope is mounted. The cross sections of the images recorded during this scan are shown in the fourth row (*x*-*z*) of Supplementary Fig. 13a,b. The resolution attenuation characteristics illustrated by the observation of sub-diffraction-limited structures (Supplementary Fig. 13a,b) are consistent with the phenomena achieved under the first experimental condition described in the Supplementary Note 2, in which the Δz_1 is set to be constant (Fig. 8c,d). We further examine a grid with features larger than the diffraction limit (Supplementary Fig. 13d) with experimental conditions identical to the study of sub-diffraction-limited structures and obtain the first imaging characteristic of the microsphere. Thus, the distance between the microsphere and the structures larger than the diffraction limit enabling high quality imaging are larger than the case of resolving sub-diffraction-limited structures. The normalized intensity of the profiles extracted from these recorded images (Supplementary Fig. 13g,h) shows that the noise signal plays a significant role in the sub-diffraction-limited imaging and that the structural information gradually became indistinguishable from the noise signal as Δz increased. However, the noise signal has negligible influence on the imaging of structures larger than the diffraction limit. As we have demonstrated, the focal length or the magnification of microspheres obtained from observing structures larger or smaller than the diffraction limit are consistent (Supplementary Fig. 10 and 11). Thus, we believe that the imaging optical paths of the microspheres corresponding to imaging samples with different size are the same. Therefore, the secondary characteristic of sub-diffraction-limited imaging using microsphere superlenses is that the signal consisting of structural information is weaker than that for structures larger than the diffraction limit and easily influenced by the noise. In the case of microsphere-based super-resolution imaging, the signal (I) recorded by the microscope or the camera can be regarded as a superposition of the propagation light (I_p), containing information about features larger than the diffraction limit, and the “converted propagation light” (I_{cp}), carrying sub-diffraction-limited structural characteristics. This is the case because the sub-diffraction-limited structural information is transmitted to the far field for imaging after passing through microsphere superlenses; i.e., $I = I_p + I_{cp}$. The intensity difference between two selected points (see the left image of Supplementary Fig. 14a) is given as follows:

$$\Delta I = I^2 - I^1 = I_{\mathrm{p}}^2 + I_{\mathrm{cp}}^2 - \left(I_{\mathrm{p}}^1 + I_{\mathrm{cp}}^1\right) = I_{\mathrm{p}}^2 - I_{\mathrm{p}}^1 + I_{\mathrm{cp}}^2 - I_{\mathrm{cp}}^1 = \Delta I_{\mathrm{p}} + \Delta I_{\mathrm{cp}} \quad (1)$$

where ΔI_{p} is induced by the propagation light and ΔI_{cp} is caused by the converted propagation light. ΔI_{p} can be extracted by analyzing results obtained from imaging without the help of the microsphere because only the propagation light carrying structural information larger than the diffraction limit is recorded for imaging in this case. As shown in Supplementary Fig. 14b, the normalized intensity of a profile in the area where the sub-diffraction-limited structures exist is in the noise level when observed without the microsphere, i.e., ΔI_{p} is in the noise level. Therefore, $\Delta I \approx I_{\mathrm{cp}}^2 - I_{\mathrm{cp}}^1 = \Delta I_{\mathrm{cp}}$, by which we can extract the effect of the converted propagation light from the recorded images observed using microspheres. After normalization, the influence of Δz on the converted propagation signal under different illumination conditions is shown in Supplementary Fig. 14c,d,f,g. The third characteristic of sub-diffraction-limited imaging based on microspheres is that the converted propagation signal carrying sub-diffraction-limited structural information decays exponentially with Δz. As a control, we also process the results of observing the grid with structures larger than the diffraction limit (see the right image of Supplementary Fig. 14a). However the exponential attenuation does not appear (Supplementary Fig. 14e,h) because ΔI_{p} plays a dominant role. Since the characteristic of exponential decay is a distinctive feature to distinguish the evanescent and propagating waves[24,25] and the super-resolution capability of the microsphere can be maintained only in the near field and degrades with the separation between the microsphere and sample (Fig. 8c,d, Supplementary Fig. 4a,b and Supplementary Fig. 13), we believe the super-resolution capability of microsphere superlenses originates from the participation of evanescent waves.

The evanescent waves can participate in the microsphere-based imaging in two or more possible manners: (1) evanescent waves generated on the sample surface containing sub-diffraction-limited structural information are directly coupled into the microsphere for super-resolution imaging[17]. This study has been conducted by the exact numerical solution of Maxwell’s equations in the cases of the incoherent imaging of point dipoles[21,26,27]. The point-dipole field can be decomposed into propagating and evanescent parts[28]. If these evanescent parts can be directly or effectively coupled into microspheres for super-resolution imaging, the exact numerical solution of Maxwell’s equations is expected to explain the experimentally observed resolution. However, the observed resolution (λ/6.3 – λ/8.4) in our study and others[5] has not been reproduced. (2) A light field containing evanescent waves can transfer the large spatial frequencies of samples into the propagating region supported by the optical transfer function[29]. Based on this mechanism, super-resolution wide-field imaging under the illumination of evanescent light generated in total internal reflection at a microfiber[30] or prism[31] surface has been

demonstrated. As incident light illuminates a microsphere, evanescent waves are generated around the shadow-side surface[13,22,32]. Specifically, the evanescent waves are generated from the second refraction of light on the microsphere surface[33], which can be observed in the FDTD simulation results calculated under partial boundary illumination (PBI, Supplementary Fig. 15c) and partial and inclined illumination (PII, Supplementary Fig. 15d) conditions (see the rectangular marked areas shown in Supplementary Fig. 15k and l). This observation is further confirmed by the exponential decay characteristic of the electric field intensity along the z direction near the microsphere apex area (Supplementary Fig. 15n). This exponential decay characteristic obtained from the FDTD simulation is consistent with the experimental observations (Supplementary Fig. 14c,d,f,g); i.e., all of them follow a similar exponential decay trend (with exponential decay coefficients in the range of 2.42 – 2.78). It has also been demonstrated that the microsphere can collect the propagating waves transferred by evanescent light generated in total internal reflection for nanoscale imaging[34]. In the following analyses, we link the evanescent waves generated by microspheres together with the converted propagation waves that will be further collected by the microsphere for super-resolution imaging.

Assuming the evanescent wave is generated by microspheres consisting of a pair of spatial frequencies $\mathbf{k}_{\parallel} = \pm\left(k_x, k_y\right)$, the transverse wavevectors of the sample $\mathbf{k}_{\parallel}^{\mathrm{s}}$ will be shifted as $\kappa_{\parallel}^{\mathrm{s}} = \mathbf{k}_{\parallel}^{\mathrm{s}} \pm \mathbf{k}_{\parallel}$. The converted/shifted waves will be observable by SSUM if $\left|\kappa_{\parallel}^{\mathrm{s}}\right| \le k_{\mathrm{max}} = 2\pi n_{\mathrm{s}}\,\mathrm{NA}/\lambda$,[29] where k_{max} is the maximum spatial frequency that can be detected by SSUM considering the solid-immersion effect[5,6]. Based on the spectral analysis, the radial frequencies of the field near the microsphere can be as high as $2k_0$ when $\Delta z < \lambda/2$,[13] where $k_0 = 2\pi n_{\mathrm{w}}/\lambda \approx 0.71k_{\mathrm{max}}$ and n_{w} is the refractive index of water. The evanescent waves with radial frequencies in the range of k_0 – $2k_0$ can extend resolution by a factor of 1.71 – 2.42, which implies that the resolution of the microsphere can be enhanced to $\lambda/6.5$ – $\lambda/9.2$ when the evanescent waves are considered. This finding is consistent with our observed range of $\lambda/6.3$ – $\lambda/8.4$ (Supplementary Note 1). Usually, the evanescent wave intensity with quite a large frequency is negligible compared with the propagating waves[13] or the background signal, which limits the microsphere from achieving its theoretical resolution limit in practice. Since the waves with higher frequencies can be generated in more limited values of Δz and under larger refractive index contrast[13], the resolution of SSUM will not be theoretically limited to $\lambda/9.2$. However, when moving away from the microsphere apex (corresponding to an increase in Δz), the higher the frequency is, the faster the evanescent wave attenuates, and the narrower the spectrum the field contains. Thus, the resolution degrades with Δz, which is consistent with our observed results shown in Fig. 8c,d, Supplementary Fig. 4a,b and Supplementary Fig. 13a,b.

Increasing the illumination intensity can directly enhance the generated evanescent field. However, this enhancement is accompanied with the negative effect that the propagating field is also promoted. Therefore, a direct way to enhance the resolution or to approach the theoretically estimated resolution is to increase the incident light intensity while decreasing the effect induced by the propagation components of the illumination or the background signal. Experimentally, the coaxial illumination (CI, Supplementary Fig. 15a) can be separated into middle illumination (MI, Supplementary Fig. 15b) and PBI (Supplementary Fig. 15c). Comparing the results shown in Supplementary Fig. 12m with the observed Blu-ray image under MI (Supplementary Fig. 12g,j and Supplementary Fig. 15f), only the structures larger than the diffraction limit can be clearly resolved under MI. Therefore, in the case of resolving sub-diffraction-limited structures, the MI mainly contributes to the background, which degrades the imaging quality. The propagating waves play a dominant role in the MI. As the signal intensity of the shifted sample high spatial frequency components by evanescent waves is submerged in the background noise, the resolution degrades (Supplementary Fig. 12f,h,i,k and Supplementary Fig. 15e,g). In addition, the PBI guarantees the sub-diffraction-limited resolution capability of the microsphere superlenses (see Supplementary Fig. 15e-g). This finding is also supported by the consistence of the exponential decay between the simulated electric field generated by PII (Supplementary Fig. 15l,n) and experimental observations (Supplementary Fig. 14c,d,f,g). Furthermore, inclining the illumination (PBI) can shift the second refraction position where the evanescent waves are generated to the middle area around the microsphere apex (Supplementary Fig. 15k,l). Correspondingly, the imaging area also shifts from the boundary area to the middle area of the microsphere, where the evanescent waves are generated, and the imaging quality also improves (Supplementary Fig. 15g,h). As the second refraction position is transferred to the microsphere apex, the areas generating evanescent waves become closer to the sample, which shortens the distance spanned by evanescent waves transferring to the sample. This decrease therefore induces a spectrum extension of the evanescent waves illuminating the sample since the evanescent waves with higher frequency can propagate to the sample before being attenuated below the background noise level. Therefore, the lack of deterioration from the MI and the transfer of the position where the evanescent waves are generated enable a larger Δz to achieve high-quality imaging under PII conditions (see Figs. 7, 8 and Supplementary Figs. 4a,b and 13). A higher resolution at the same Δz under PII conditions can also be expected as the evanescent waves with higher spatial frequencies induced by the spectrum extension shift the larger spatial frequencies of the specimen into the propagating field[29] and the negative effect of MI is excluded (see Fig. 8c,d and Supplementary Fig. 4a,b).

Therefore, we believe that (1) the interaction of the sample and the evanescent waves generated from the second refraction on the microsphere surface plays a role in the origin of the sub-diffraction-limited resolution of the microsphere superlens. Recently, Alexey

V. Maslov and Vasily N. Astratov demonstrated that the excitation of samples' electromagnetic modes plays an important role in optical images of sub-wavelength structures[19], which provides a possible interpretation of the influence of the light field generated by microspheres on the sample and an extraordinary high resolution accompanied with artifacts that may distort the optical images in experimental observations[35]. (2) The imaging quality under PII is higher than that under CI and Δz enabling high-quality imaging for PII larger than CI owing to the loss of deterioration from the MI that mainly introduces propagating light in imaging and the transfer of the second refraction position by inclining illumination which shortens the distance for evanescent waves transferring to the sample and induces a spectrum extension of the evanescent waves illuminating the sample surface.

Supplementary References

1. Sparrow, C. M. On spectroscopic resolving power. *Astrophys. J.* **44**, 76-86 (1916).
2. Abbe, E. Beiträge zur Theorie des Mikroskops und der mikroskopischen Wahrnehmung. *Archiv für mikroskopische Anatomie* **9**, 413-418 (1873).
3. Houston, W. V. A compound interferometer for fine structure work. *Phys. Rev.* **29**, 0478-0484 (1927).
4. Rayleigh, L. XXXI. Investigations in optics, with special reference to the spectroscope. *The London, Edinburgh, and Dublin Philosophical Magazine and Journal of Science* **8**, 261-274 (1879).
5. Allen, K. W. *et al.* Super-resolution microscopy by movable thin-films with embedded microspheres: Resolution analysis. *Ann. Phys.-Berlin.* **527**, 513-522 (2015).
6. Allen, K. W. *et al.* Overcoming the diffraction limit of imaging nanoplasmonic arrays by microspheres and microfibers. *Opt. Express* **23**, 24484-24496 (2015).
7. Darafsheh, A., Limberopoulos, N. I., Derov, J. S., Walker Jr, D. E. & Astratov, V. N. Advantages of microsphere-assisted super-resolution imaging technique over solid immersion lens and confocal microscopies. *Appl. Phys. Lett.* **104**, 061117 (2014).
8. Darafsheh, A., Walsh, G. F., Dal Negro, L. & Astratov, V. N. Optical super-resolution by high-index liquid-immersed microspheres. *Appl. Phys. Lett.* **101**, 141128 (2012).
9. De Graef, M. *Introduction to conventional transmission electron microscopy*, (Cambridge University Press, 2003).
10. Orloff, J. *Handbook of charged particle optics*, (CRC press, 2008).
11. Rai-Choudhury, P. *Handbook of microlithography, micromachining, and microfabrication: microlithography* Vol. 1, (SPIE Press, 1997).
12. Darafsheh, A., Guardiola, C., Palovcak, A., Finlay, J. C. & Cárabe, A. Optical super-resolution imaging by high-index microspheres embedded in elastomers. *Opt. Lett.* **40**, 5-8 (2015).
13. Devilez, A., Stout, B., Bonod, N. & Popov, E. Spectral analysis of three-dimensional photonic jets. *Opt. Express* **16**, 14200-14212 (2008).
14. Hecht, E. *Optics* 4th edn, (Addison-Wesley, 2002).

15. Wang, Z. B. *et al.* Optical virtual imaging at 50 nm lateral resolution with a white-light nanoscope. *Nat. Commun.* **2**, 218 (2011).
16. Heifetz, A., Simpson, J. J., Kong, S. C., Taflove, A. & Backman, V. Subdiffraction optical resolution of a gold nanosphere located within the nanojet of a Mie-resonant dielectric microsphere. *Opt. Express* **15**, 17334-17342 (2007).
17. Li, L., Guo, W., Yan, Y., Lee, S. & Wang, T. Label-free super-resolution imaging of adenoviruses by submerged microsphere optical nanoscopy. *Light-Sci. Appl.* **2**, e104 (2013).
18. Regan, C. J., Rodriguez, R., Gourshetty, S. C., de Peralta, L. G. & Bernussi, A. A. Imaging nanoscale features with plasmon-coupled leakage radiation far-field superlenses. *Opt. Express* **20**, 20827-20834 (2012).
19. Maslov, A. V. & Astratov, V. N. Imaging of sub-wavelength structures radiating coherently near microspheres. *Appl. Phys. Lett.* **108**, 051104 (2016).
20. Regan, C. J., Dominguez, D., de Peralta, L. G. & Bernussi, A. A. Far-field optical superlenses without metal. *J. Appl. Phys.* **113** (2013).
21. Duan, Y. B., Barbastathis, G. & Zhang, B. L. Classical imaging theory of a microlens with super-resolution. *Opt. Lett.* **38**, 2988-2990 (2013).
22. Heifetz, A., Kong, S.-C., Sahakian, A. V., Taflove, A. & Backman, V. Photonic nanojets. *J. Comput. Theor. Nanos.* **6**, 1979 (2009).
23. Li, X., Chen, Z., Taflove, A. & Backman, V. Optical analysis of nanoparticles via enhanced backscattering facilitated by 3-D photonic nanojets. *Opt. Express* **13**, 526-533 (2005).
24. Mattheyses, A. L. & Axelrod, D. Direct measurement of the evanescent field profile produced by objective-based total internal reflection fluorescence. *J. Biomed. Opt.* **11** (2006).
25. Sarkar, A., Robertson, R. B. & Fernandez, J. M. Simultaneous atomic force microscope and fluorescence measurements of protein unfolding using a calibrated evanescent wave. *P. Natl. Acad. Sci. USA* **101**, 12882-12886 (2004).
26. Hoang, T. X., Duan, Y. B., Chen, X. D. & Barbastathis, G. Focusing and imaging in microsphere-based microscopy. *Opt. Express* **23**, 12337-12353 (2015).
27. Sundaram, V. M. & Wen, S.-B. Analysis of deep sub-micron resolution in microsphere based imaging. *Appl. Phys. Lett.* **105**, - (2014).
28. Setala, T., Kaivola, M. & Friberg, A. T. Decomposition of the point-dipole field into homogeneous and evanescent parts. *Phys. Rev. E* **59**, 1200-1206 (1999).
29. Novotny, L. & Hecht, B. *Principles of Nano-Optics*, (Cambridge Univ. Press, 2012).
30. Hao, X. *et al.* Far-field super-resolution imaging using near-field illumination by micro-fiber. *Appl. Phys. Lett.* **102**, 013104 (2013).
31. Hao, X., Kuang, C. F., Li, Y. H. & Liu, X. Evanescent-wave-induced frequency shift for optical superresolution imaging. *Opt. Lett.* **38**, 2455-2458 (2013).
32. Chen, Z., Taflove, A. & Backman, V. Photonic nanojet enhancement of backscattering of light by nanoparticles: a potential novel visible-light ultramicroscopy technique. *Opt. Express* **12**, 1214-1220 (2004).
33. Itagi, A. V. & Challener, W. A. Optics of photonic nanojets. *J. Opt. Soc. Am. A* **22**, 2847-2858 (2005).
34. Sciammarella, C. A. Experimental mechanics at the nanometric level. *Strain* **44**, 3-19 (2008).

35. Astratov, V. N. *et al.* Label-Free Nanoscopy with Contact Microlenses: Super-Resolution Mechanisms and Limitations. Preprint at http://arxiv.org/abs/1606.05145 (2016).
